# The *Aspergillus fumigatus* Sialidase (Kdnase) Contributes to Cell Wall Integrity and Virulence in Amphotericin B-Treated Mice

**DOI:** 10.3389/fmicb.2017.02706

**Published:** 2018-01-18

**Authors:** Jason R. Nesbitt, Elizabeth Y. Steves, Cole R. Schonhofer, Alissa Cait, Sukhbir S. Manku, Juliana H. F. Yeung, Andrew J. Bennet, Kelly M. McNagny, Jonathan C. Choy, Michael R. Hughes, Margo M. Moore

**Affiliations:** ^1^Department of Biological Sciences and the Centre for Cell Biology, Development and Disease, Simon Fraser University, Burnaby, BC, Canada; ^2^Biomedical Research Centre, The University of British Columbia, Vancouver, BC, Canada; ^3^Department of Molecular Biology and Biochemistry, Simon Fraser University, Burnaby, BC, Canada; ^4^Department of Chemistry, Simon Fraser University, Burnaby, BC, Canada

**Keywords:** invasive aspergillosis, sialidase, cell wall integrity, chitin, Kdn

## Abstract

*Aspergillus fumigatus* is a filamentous fungus that can cause a life-threatening invasive pulmonary aspergillosis (IPA) in immunocompromised individuals. We previously characterized an *exo*-sialidase from *A. fumigatus* that prefers the sialic acid substrate, 2-keto-3-deoxy-D-*glycero*-D-*galacto*-nononic acid (Kdn); hence it is a Kdnase. Sialidases are known virulence factors in other pathogens; therefore, the goal of our study was to evaluate the importance of Kdnase in *A. fumigatus*. A *kdnase* knockout strain (*Δkdnase*) was unable to grow on medium containing Kdn and displayed reduced growth and abnormal morphology. *Δkdnase* was more sensitive than wild type to hyperosmotic conditions and the antifungal agent, amphotericin B. In contrast, *Δkdnase* had increased resistance to nikkomycin, Congo Red and Calcofluor White indicating activation of compensatory cell wall chitin deposition. Increased cell wall thickness and chitin content in *Δkdnase* were confirmed by electron and immunofluorescence microscopy. In a neutropenic mouse model of invasive aspergillosis, the *Δkdnase* strain had attenuated virulence and a significantly lower lung fungal burden but only in animals that received liposomal amphotericin B after spore exposure. Macrophage numbers were almost twofold higher in lung sections from mice that received the *Δkdnase* strain, possibly related to higher survival of macrophages that internalized the *Δkdnase* conidia. Thus, *A. fumigatus* Kdnase is important for fungal cell wall integrity and virulence, and because Kdnase is not present in the host, it may represent a potential target for the development of novel antifungal agents.

## Introduction

*Aspergillus fumigatus* is a filamentous fungus that can cause a life-threatening systemic mycosis called invasive aspergillosis (IA). IA cases are rare in immunocompetent patients but affect those with weakened immune systems caused by immunosuppressive drug treatment, cancer or genetic polymorphisms that affect the innate immune response (Schmiedel and Zimmerli, 2016). IA typically begins in the lungs after inhalation of airborne fungal conidiospores (conidia) that attach to the epithelium and germinate. If the epithelial barrier is breached, the fungus may disseminate hematogenously to other organs such as the brain or kidneys (Young et al., 1970). Other less common portals of entry for invasive *Aspergillus* infection include the skin, gastrointestinal tract, and the eyes (Fuqua et al., 2010). IA is usually treated with the triazole antifungal, voriconazole as the primary treatment, and the polyene antibiotic, amphotericin B as an alternative. Caspofungin, an echinocandin antifungal agent, is utilized as a salvage therapy as it is generally less toxic than the other two drugs (Walsh et al., 2008; Kousha et al., 2011). Despite drug treatment, the overall mortality rate for IA is approximately 50% and other factors can worsen prognosis, such as the progression of underlying malignancy, renal impairment, or recent corticosteroid treatment (Nivoix et al., 2008).

There are more than 250 species in the *Aspergillus* genus. Most invasive infections are caused by members of the *A. fumigatus* species complex followed by organisms in the *A. terreus*, *A. flavus*, and *A. niger* complexes (Lass-Florl and Cuenca-Estrella, 2017). Fungal pathogenesis is complex and requires the normally saprotrophic *Aspergillus* spp. to adapt to the environment of the human lung. Numerous excellent reviews have summarized the pathways or molecules that contribute to the ability of *A. fumigatus* to colonize and invade host tissue including transcription factors (Bultman et al., 2017), secondary metabolites such as toxins or siderophores (Scharf et al., 2014) and immune evasion factors (Chotirmall et al., 2014). Binding to host tissues is an important first step in establishing an infection. *A. fumigatus* secretes the polysaccharide, galactosaminogalactan (GAG) that has been implicated in both adhesion to host tissue (biofilm formation) and virulence (Gravelat et al., 2013). GAG also contributes to immunosuppression by inducing apoptosis of neutrophils (Robinet et al., 2014).

Adhesion of *A. fumigatus* conidia to the host is also mediated by sialic acids. Sialic acids are a family of >50 derivatives of the 9-carbon monosaccharide, neuraminic acid. Two naturally occurring sialic acids have a substitution at carbon 5 with an *N*-acetyl or an –OH group yielding *N*-acetylneuraminic acid (Neu5Ac) or 2-keto-3-deoxy-D-*glycero*-D-*galacto*-nononic acid (Kdn), respectively (Varki and Schauer, 2009). We demonstrated previously that fungal sialic acids were responsible, in part, for binding of *A. fumigatus* conidia to fibronectin and intact basal lamina and that *A. fumigatus* contained higher levels of sialic acid on the conidial surface compared to non-pathogenic species (Wasylnka and Moore, 2000; Wasylnka et al., 2001). Enzymatic removal of sialic acids on conidia led to a decrease in their uptake by both cultured murine macrophages and cultured type 2 pneumocytes (Warwas et al., 2007). Sialic acids have also been detected on numerous other species of pathogenic fungi including *Fonsecaea pedrosoi*, *Sporothrix schenckii, Paracoccidioides brasiliensis, Cryptococcus neoformans*, and *Candida albicans* (Alviano et al., 1999).

The sialic acid biosynthetic pathway has not been identified in any fungus to date; however, we have previously identified and characterized a sialidase from *A. fumigatus*. Sialidases are glycoside hydrolase enzymes that catalyze the release of sialic acids from glycans present on the cell surface or in their environment. The *A. fumigatus* sialidase has an N-terminal secretion sequence and may therefore have a role in modifying cell wall carbohydrates and/or cleaving extracellular carbohydrates. Recombinant *Af*sialidase cleaved terminal synthetic substrates with α2,3-linked sialic acids to a greater extent that those with α2,6-linked sialic acids; hydrolysis of 2,8-linked sialic acids by the *A. fumigatus* sialidase was also demonstrated (Warwas et al., 2010). Growth in medium containing human serum induced sialidase gene expression (Warwas et al., 2010). Subsequent studies revealed that the *A. fumigatus* sialidase activity was not inhibited by the classical sialidase inhibitor, 2-deoxy-2,3-didehydro-N-acetylneuraminic acid (DANA) (Telford et al., 2011). Furthermore, the crystallized *A. fumigatus* sialidase covalently bound 3-F-β-Kdn in the enzyme active site. Kinetic analysis showed that the catalytic efficiency (*k*_cat_*/K*_m_) was 5 orders of magnitude higher with Kdn-methylumbelliferone compared to the Neu5Ac substrate (Telford et al., 2011). Hence, the *A. fumigatus* sialidase is a Kdnase but its importance to survival of *A. fumigatus in vitro* and *in vivo* is unknown.

Sialidases are involved in the virulence of several mammalian pathogens. For example, viral sialidase is critical for completing the life cycle of influenza virus as it prevents viral recapture upon budding (Palese et al., 1974). Sialidase activity is also important for the virulence of several bacterial pathogens. For example, *S. pneumoniae* expresses three sialidases: NanA, NanB, and NanC, and a *ΔnanA* mutant of the R6 cell line displayed reduced adherence to D562 human pharyngeal cells and a decrease in biofilm formation (King et al., 2006). Recent evidence has revealed that internalization of *Salmonella* ser. Typhimurium is dependent on glycoside hydrolases including the sialidase, NanH. Deletion of *nanH* resulted in a decrease of adhesion and invasion of Caco-2 cells equivalent to the level observed with mutants of the Type III secretion system (T3SS) (Arabyan et al., 2016)

The goals of the present study were to determine whether the *Aspergillus fumigatus* sialidase (Kdnase) is critical for the growth and development of *A. fumigatus* and whether it is required for virulence. Specifically, we examined the phenotypes and cell wall carbohydrates of the parental, Kdnase mutant; and, complemented strains grown in various carbon sources and under conditions of cell wall stress, including the effect of antifungal agents. The interaction of conidia with macrophages *in vitro* was also investigated. Finally, we determined the relative virulence of the strains in a neutropenic mouse model of IA.

## Materials and Methods

### *A. fumigatus* Strains and Growth Conditions

*Aspergillus fumigatus* strain ATCC 13073 (American Type Culture Collection, Manassas, VA, United States), originally isolated from a human pulmonary lesion, was used throughout this study as the wild type (WT) or parental strain. Unless stated otherwise, fungi were grown at 37°C on yeast extract-agar-glucose (YAG, 0.5% yeast extract, 2% glucose, 0.125% MgSO_4_∙7H_2_O, 0.1% Hutner’s trace elements, 0.1% Vitamins, 1.5% agar) or Kafer’s minimal medium (Hill and Kafer, 2001) supplemented with Hutner’s trace elements and vitamins. All strains were stored on silica gel at -80°C.

### Harvesting Conidia

Mature conidia were harvested by flooding an agar plate with phosphate-buffered saline, pH 7.4 supplemented with 0.05% Tween 20 (PBST). The surface was gently agitated with a sterile cotton swab and the suspension was filtered through sterile Miracloth (Calbiochem) to remove hyphal fragments. Conidia were then washed three times in sterile PBS. Spores were counted with a haemocytometer.

### Fungal Growth on Varying Carbon Sources or Stressors

Kafers minimal media with a 1% Hutner’s vitamin mixture was used to compare the growth of wild type and Δ*kdn*ase *A. fumigatus* strains. The carbon source was changed as indicated. Equivalent numbers of conidia were inoculated into the center of each plate and plates were incubated for 72 h at 37°C. Hygromycin (100 μg/mL) was the positive selection marker. To test the effect of sorbitol on growth, YAG solid media plates (with or without 1 M sorbitol) were inoculated with 10^5^ conidia, in a volume of 5 μL in the center of each plate. Slide cultures were also prepared for each strain on YAG supplemented with 750 μg/mL Congo Red dye and incubated for 20 h at 37°C. Images were obtained on a Zeiss inverted microscope at 100× magnification.

### *A. fumigatus* Growth Assays in the Presence of Antifungal Drugs

Growth assays were prepared and monitored following the EUCAST protocol (EUCAST E.DEF 9.3 December 2015) modified by addition of resazurin to increase the sensitivity of growth measurement (Yamaguchi et al., 2002). Assays were prepared in 96-well microdilution plates with RPMI 1640 media buffered with 3-(*N*-morpholino)propanesulfonic acid (MOPS) at a final concentration of 0.165 M, pH 7.0, and 2 × 10^5^ conidia/mL. Antifungal compounds were prepared in dimethyl sulfoxide (DMSO) to a maximum of 2.5% v/v. Control wells contained DMSO alone. Resazurin stock solution (Sigma–Aldrich) was prepared in PBS and filter sterilized, and media were prepared with a final concentration of 0.02% resazurin. Plates were incubated in the dark at 37°C. Growth was quantified in a SpectraMax plate reader by measuring resorufin fluorescence after 36–48 h of incubation (excitation wavelength 560 nm; emission wavelength 590 nm). Control plates were done in parallel that contained all components except conidia confirmed that antibiotics did not affect resorufin fluorescence.

### Construction and Confirmation of *kdnase* Knockout Strain and Complemented Strain

The *kdnase* knockout was created by homologous recombination using a disruption construct containing 1000 bp sequences of DNA encoding the upstream and downstream regions of the *kdnase* gene, flanking a positive selectable marker for hygromycin resistance (Supplementary Figure S1). The disruption construct was generated using fusion PCR and introduced into the wild type strain using *Agrobacterium*-mediated transformation as follows. The hygromycin resistance gene cassette containing the *Escherichia coli* (hygromycin phosphotransferase, *hph*) under the control of the constitutive *trpC* promoter and terminator from *Aspergillus nidulans* (Hamert and Timberlaket, 1987) was amplified with the primers 5′- AAACATTCCCACTATCGCGA TCGTCGACGTTAACTGATATTGA and 5′-TAATGCTAGTCCAGCAACGACGTCGAC GTTAACTGGTTCC. The upstream and downstream 1000 base pairs of DNA that flank the *kdn*ase gene in were amplified by PCR with the following primer pairs, upstream: 5′- GCGCTCCTATCCAGTCAGTC; 5′-TCGCGATAGTGGGAATGTTT and downstream: 5′- TCGTTGCTGGACAGCATTAT; 5′-TGCTTCATGTCATGCCTAGC. The *hph* cassette and *kdnase* flanking regions were amplified by PCR using iProof^TM^ High-fidelity DNA polymerase (Bio-Rad, Mississauga, ON, Canada). Fusion PCR was performed with high-fidelity AccuPrime *Taq* DNA polymerase (Thermo Fisher Scientific, Mississauga, ON, Canada) using the primers 5′-GGGATCCACATACCATTCTCGCCGAAC and 5′-GGGATCCAATGCTA CGGGAACACTTGG that added a *BamH1* restriction site to facilitate insertion into the pCambia plasmid. The fusion PCR fragment was gel purified and cloned into pCambia 0380 plasmid (Cambia) yielding pCambia-KdnaseKO. Competent *Agrobacterium tumefaciens* (AGL-1) were transformed using heat shock. Transformation of *A. fumigatus* followed the protocol of Sánchez and Aguirre (1996) with modifications. Putative fungal transformants were harvested using a punch transfer tool and placed on fresh YAG agar plates containing 100 μg/mL hygromycin. Plates were incubated at 37°C for 72 h before mature spores were harvested. Conidial suspensions were then diluted and re-plated with an estimated 10 spores per plate to generate plates with colonies grown from single spores.

*XhoI*-digested DNA from the WT and mutant strains was analyzed by Southern blotting to ensure that only a single copy of the *kdnase* gene was integrated into the genome and that the integration was at the *kdnase* locus (Supplementary Figure S2). The knockout strain was also sequenced at the *kdnase* locus to ensure that up- and downstream sequences were not affected. Successful homologous integration of the knockout construct in the putative mutants was also confirmed by PCR (Supplementary Figure S3).

To construct *Δkdnase^R^*, the rescued strain, iProof polymerase (Bio-Rad) was used to amplify 2470 base pairs of the wild type *kdnase* gene plus 928 bp of upstream sequence [including TATA and CAAT boxes (Warwas et al., 2010)] and 322 bp of downstream sequence.

The rescue construct was amplified with the primers 5′-TGCAAGCTTCAGA ACTGCCCTTTCCCTCT-3′ and 5′-GTTAAGCTTACTGTTTTGCTGCCCTCTTC-3′ containing *HindIII* restriction sites to facilitate insertion into pBCPhleo (Silar, 1995). pBC-phleo-Kdnase was used to transform *E. coli* DH5α and plasmid DNA was linearized with *SpeI* (New England Biolabs, Whitby, ON, Canada). Linearized plasmid DNA (10 μg) was introduced into 50 μL of swollen conidia by electroporation. Briefly, ice-cold swollen conidia were pelleted by centrifugation and washed 3 times with 4°C sterile water after which they were re-suspended in 4 mL YED media (1% yeast extract, 1% glucose, 20 mM HEPES, pH 8) and incubated for 1 h at 30°C with gentle shaking. Following incubation, conidia were centrifuged and re-suspended in 150 μl of electroporation buffer (10 mM Tris–HCL pH7.5, 270 mM sucrose, and 1 mM lithium acetate). The conidial suspension was then divided into 50 μl aliquots for use in transformation. The restriction enzyme *SpeI* (New England Biolabs) was used to linearize pBC-phleo-Kdnase. Linearized plasmid DNA (10 μg) was added to 50 μl of prepared conidia and the mixture was incubated for 15 min on ice before being transferred to an electroporation cuvette with a 2 mm gap. Conidia were exposed to an electric field pulse of 1 kV, 400 ohms, and 25 μF. Immediately following the pulse, 1 mL of ice-cold YED media was added to the conidia and they were cooled on ice for 15 min before being allowed to recover for 2 h at 37°C with gentle shaking. Transformants were selected on YAG media containing 100 μg/mL phleomycin. Putative transformants were subcultured onto selective media to obtain pure cultures. Ectopic integration of the rescue construct was confirmed using PCR (Supplementary Figure S3).

### Semi-quantitative Analysis of Cell Wall Polysaccharides

#### α(1,3)-Glucan and β(1,3)-Glucan

Sterile glass cover slips were dried and added to Petri dishes containing 20 ml YAG with or without 1 M sorbitol. Conidia of the appropriate strain were added and allowed to germinate overnight at 37°C. Conidia incubated in medium containing sorbitol required an additional 6 h of growth. Coverslips were removed and washed with PBS and prepared for immunofluorescence microscopy as follows: for α(1,3)-glucan analysis, a mouse monoclonal antibody specific to α(1,3)-glucan (Sigma, M5170) was added to a PBS solution containing 0.5% BSA and 0.05% Tween 20 at a dilution of (1:10) and slides were incubated in the dark overnight at 4°C. After washing with PBS, an AlexaFluor 488-conjugated secondary antibody (Jackson ImmunoResearch, 1:50 dilution) was added and the coverslips were incubated for 1 h at room temperature. Coverslips were mounted on slides after rinsing with PBS and adding Prolong Gold anti-fade solution. Slides were allowed to dry, sealed with nail polish and stored at 4°C in the dark. Fluorescence was visualized on a spinning disk confocal microscope (WaveFX spinning disk confocal system, Quorum Technologies) and signals were quantified using Volocity 6.3 imaging software (PerkinElmer). Samples for β(1,3)-glucan analysis were treated identically except that the monoclonal antibody was obtained from BioSupplies, Australia (mouse monoclonal antibody to β(1,3)-glucan) (IgG, Kappa Light; Cat. No. 400-2).

#### Chitin

Fungi on coverslips were incubated with 50 μL of a chitin probe solution (TMR-Star Conjugated Chitin-binding Probe, a gift from New England Biolabs Ltd.) (20 μg/mL in PBS) overnight in the dark at 4°C. Coverslips were rinsed with PBS, dried and processed as described for α(1,3)-glucan staining. Fluorescence was visualized on a spinning disk confocal microscope (WaveFX spinning disk confocal system, Quorum Technologies) and signals were quantified using Volocity 6.3 imaging software (PerkinElmer). For each strain/polysaccharide, at least 30 images were analyzed and exposure was controlled manually at 900 ms. Control samples were processed in parallel except that the primary antibody or chitin probe was not added.

### Transmission Electron Microscopy (TEM)

Petri dishes containing YAG agar or YAG + 1M sorbitol agar were prepared and overlaid with a sterile disk of cellophane. Conidia of the appropriate fungal strain (1 × 10^6^) were spread evenly over the surface of the cellophane. Each plate was incubated at 30°C for 20 h than at 37°C for 5 h and flooded with a fixative solution containing 2.5% EM-grade glutaraldehyde in 0.1 M sodium cacodylate buffer, pH 7.4. Plates were incubated at room temperature for 1 h, washed with sodium cacodylate buffer and a 1-cm^2^ piece of cellophane was transferred to a microcentrifuge tube filled with 0.1 sodium cacodylate buffer. Samples were dehydrated in an ethanol series, followed by 1:1 solution of ethanol/propylene oxide (1:1) then pure propylene oxide. The samples were soaked in a series of propylene oxide/Spurr resin mixtures (3:1, 1:1, 1:3, 0:1). Each sample was incubated in 100% resin for 2 days with resin replaced twice each day. Resin was polymerized at 70°C for 8 h. Samples were examined on a Hitachi H-7600 transmission electron microscope. From the TEM images of hyphae, cell wall thickness was quantified with ImageJ using the measure function. A random number generator produced 5 sets of x,y co-ordinates for each image. ImageJ was used to locate coordinates and measure the thickness of the cell wall directly across using the measurement tool (calibrated to each scale bar). If the chosen co-ordinate was not on the cell wall, the line tool was used to find the shortest straight line to the cell wall, and the wall measurement was made at that point where the angle of intersection with each side of the cell wall as close to 90 degrees as possible. Measurements were performed blinded on coded images. The number of separate images analyzed for each condition was as follows: WT (17), Δ*kdnase* (17), WT + 1M sorbitol (12), Δ*kdnase* + 1 M sorbitol (24).

### Scanning Electron Microscopy

Conidia (5 × 10^6^) from each strain were incubated in RPMI 1640 overnight at 37°C on sterile round coverslips (25 mm). RPMI was replaced with 3 mL of a solution containing 0.075% Alcian Blue, 75 mM lysine, 3% glutaraldehyde in 0.1 M sodium cacodylate buffer, pH 7.2 and further incubated at 4°C overnight. Addition of Alcian Blue and lysine have been shown to enhance visualization of the polyanionic exopolysaccharides (Fischer et al., 2005). Samples were washed 3 times each with 0.1 M sodium cacodylate buffer, pH 7.2, and distilled water. After dehydration in ethanol (25, 50, 75, 90, 100, and 100% for 10 min each), samples were critical point dried, coated with iridium (5 ± 1 nm) and examined on a Nova NanoSEM (FEI).

### Neutropenic Mouse Model of Invasive Aspergillosis

All experiments were carried out in accordance with the Canadian Council on Animal Care (CCAC) guidelines. This study was approved by the University Animal Care Committee at Simon Fraser University (protocol 1193B-11). The mouse model of pulmonary IA was modified from (Dixon et al., 1989). Briefly, female BALB/cJ mice (∼3 months of age) from Jackson Laboratory (Bar Harbor, ME, United States) were acclimatized for 2 weeks prior to experimentation. Mice were immunocompromised with intraperitoneal injections of 0.025 g/ml cyclophosphamide (Toronto Research Chemicals, North York, ON, Canada) in PBS (100 mg/kg) administered on day 4 and day 0, and subcutaneous injections of 0.05 g/mL cortisone acetate (Sigma–Aldrich Canada Co., Oakville, ON, Canada) in PBST (200 mg/kg) on day 1. Experimental groups were inoculated intranasally with 1.0 × 10^6^ fungal spores in 40 μL aliquots, as follows: seven mice received wild type *Aspergillus fumigatus* spores, seven mice received wild type *Aspergillus fumigatus* spores followed by a dose of 5 mg/kg of liposomal amphotericin B (AmBisome) (AB) (Gilead Sciences Inc., Foster City, CA, United States), seven mice received Δ*kdnase A. fumigatus* spores, seven mice received Δ*kdnase A. fumigatus* spores and 5 mg/kg AB, seven mice received *Δkdnase^R^* strain *A. fumigatus* spores, three mice received saline, and four mice received saline and 5 mg/kg AB. AB was administered 4 h after spore inoculation on day 0; the drug was resuspended in water just prior to use, and administered intravenously.

Mice were maintained in a specific pathogen-free facility, four to a cage, with alternating 12-h light schedules. Mice were housed in autoclaved cages and received irradiated chow *ad libitum* (equal parts mixture of PicoLab Mouse Diet 20 and Picolab Rodent Diet 20). Mice were provided with a tetracycline water (0.5 g/L) mixture sweetened with aspartame (Sigma–Aldrich Canada Co., Oakville, ON, Canada). Mice were monitored and weighed daily. At experimental end-points (>20% weight loss, swelling around the eyes, piloerection, hunched posture, limited mobility and activity, rapid breathing or gasping, and antisocial behavior), mice were humanely euthanized by CO_2_ followed by removal of lungs. After euthanasia, perfusion with ice cold PBS was performed to clear blood from mouse lungs. The left lobe was fixed overnight at 4°C in paraformaldehyde, then transferred to an ethanol solution, and prepared for histology. The right lobe was split into two, one section was stored in TRIzol (Invitrogen) at 4°C for cytokine analysis via qPCR, the other section was frozen in a pre-weighed bag at -20°C for DNA analysis for fungal burden.

### Lung Histology

Fixed lungs were sectioned and stained with hematoxylin and eosin (H&E) or with Grocott’s methenamine silver (GMS) to visualize fungal elements (hyphae and conidia). Fungal elements were quantified as total fungal element area using the color threshold tool in ImageJ on GMS-stained sections, and expressed as percent fungal element coverage of total lung area.

### Quantification of Macrophages and Neutrophils Cells in Fixed Lung Sections

Lung sections (4 μm thick) were de-paraffinized with 3 xylene washes and re-hydrated by successive washes in 100, 90, and 70% ethanol, and then finally in dH_2_O. Heat-based antigen retrieval was performed by heating slides in citrate buffer (pH 6.0) to 120°C in a pressure cooker. Slides were then incubated with 5% donkey serum for 30 min and endogenous peroxidase was inhibited with 0.3% H_2_O_2_ treatment for 20 min. Slides were incubated with the primary antibody overnight, the biotinylated secondary antibody for 30 min, and horseradish peroxidase-conjugated avidin-biotin complex (Vector Laboratories, Burlingame, CA, United States) for 45 min. Staining was visualized with 3-amino-9-ethyl carbazole (AEC) substrate chromagen, which results in red staining (Vector Laboratories). Sections were then counterstained with hematoxylin. The following primary antibodies were used: Mac-3 (5 μg/mL) (BD Biosciences, Franklin Lakes, NJ, United States) to visualize macrophages and myeloperoxidase (MPO) to visualize neutrophils (5 μg/mL) (Abcam, Cambridge, MA, United States). Immune cells were manually counted on a microscope. Quantification of the staining was performed in a manner that was blinded from knowledge of specific mouse IDs. The total lung area for each section was quantified in ImageJ and Mac-3-positive or MPO-positive cells were expressed per total area (mm^2^).

### Fungal Burden Analysis by qPCR

Analysis was based on the method of Bowman et al. (2001). Frozen lung samples in pre-weighed bags were minced and homogenized in cold saline (7.5 volumes/g lung) using a roller bottle, and transferred to microcentrifuge tubes. Secondary homogenization was carried out in a TissueLyser II (3 sets of 30 s at 30 oscillations/s) and centrifuged for 5 min at 800*g* and 4°C. Genomic DNA was extracted using the CTAB method (Carlson et al., 1991) and fungal DNA was amplified by TaqMan quantitative PCR (Life Technologies). We targeted the internal transcribed spacer region, ITS1 and the 5.8S gene located between the 18S and 28S rRNA genes, using the following primers: Forward Primer - TCTGAAAGTATGCAGTCTGAGTTGATT; Reverse Primer - GATGCCGGAACCAAGAGATC; Probe - CGTAATCAGTTAAAACTTTC. A *C*_t_ value was determined for triplicates from each sample, based on a standard curve also created using triplicate samples with genomic DNA from *A. fumigatus* (0.001–1.000 ng/μL).

### Interaction of Conidia with J774 Mouse Macrophages

To determine the proportion of conidia internalized by macrophages, we labeled conidia with fluorescein isothiocyanate (FITC) (470 nm). Bound/external conidia were detected by an anti-*Aspergillus* antibody conjugated to a red fluorophore (590 nm) added to the cell suspension at the end of the incubation period. All strains were grown on YAG media supplemented with appropriate antibiotics (100 μg/mL hygromycin for the *Δkdnase* strain and 50 μg/mL phleomycin for *Δkdnase^R^*) for 72 h at 37°C and harvested in PBST as described above. To label conidia with FITC, we followed the procedure of Sturtevant and Latge (1992). The J774A.1 cell mouse macrophage cell line, originally derived from BALB/cN mice (ATCC, Manassas, VA, United States) were grown on coverslips in 24-well plates in RPMI 1640 media containing 10% fetal bovine serum (RPMI-FBS). Macrophages (2.5 × 10^5^ cells) were incubated at 37°C + 5% CO2 for 3 h and blocked in RPMI-FBS containing 0.5% BSA for 1 h at 37°C. The medium was aspirated and FITC-labeled conidia from each strain (2 × 10^6^, suspended in 1 mL RPMI-FBS) were added to the wells. Each strain was inoculated into separate wells and no spore control and no primary antibody control wells were also included. Plates were incubated for 1 h at 37°C in 5% CO_2_ then placed on ice. On ice, J774 cells were washed 3× with PBST and incubated for 1 h with a 1:75 dilution of polyclonal rabbit anti-*Aspergillus* cell wall antibody (Wasylnka and Moore, 2002) in PBS plus 10% goat serum (GS). After washing 3 times, Alexa Fluor 594-conjugated AffiniPure goat anti-rabbit IgG (H+L) (Jackson ImmunoResearch) was added in 1:575 dilution in PBS-GS. After incubation for 1 h, cells were washed with PBS and fixed in PBS-4% formalin for 1 h. Washed coverslips were mounted with Prolong Antifade Mounting Media, sealed and stored in the dark at 4°C. Images were obtained a Zeiss Axioplan epifluorescence microscope.

Macrophage killing of conidia was measured *in vitro* using the nystatin protection assay (Wasylnka and Moore, 2002), except that J774A.1 cells were incubated in RPMI-FBS containing 0, 10, or 20 μg/mL hydrocortisone dissolved in DMSO for 24 h prior to adding conidia. Control samples were treated with DMSO.

## Results

### Deletion of the *A. fumigatus* Kdnase

The *kdnase* gene on chromosome 4 was disrupted by *Agrobacterium*-mediated transformation of *A. fumigatus* using a fusion PCR construct that contained a hygromycin resistance cassette for positive selection flanked by 1000 bp *kdnase* up- and downstream sequences (Supplementary Figure S1). Successful generation of a *Δkdnase* strain was determined by PCR and Southern blotting. The Southern blot confirmed homologous insertion of the knockout construct at the *kdnase* locus (Supplementary Figure S2). The complemented mutant, *Δkdnase^R^* or ‘rescued’ strain, was produced by electroporation of the knockout with the wild type (WT) *kdnase* gene controlled by its native promoter (928 bp of upstream sequence). Successful *kdnase* knockout and ectopic insertion of the WT *kdnase* gene into the genome of the *Δkdnase* strain was confirmed by PCR (Supplementary Figure S3).

### Comparative Growth of Wild Type and *Δkdnase* Strains on Minimal Media Supplemented with Varying Carbon Sources

**FIGURE 1:**
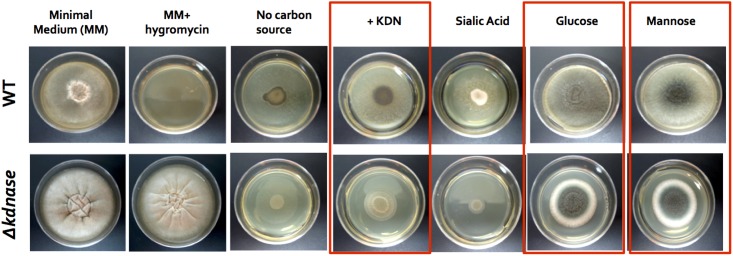
Comparative growth of wild type and Δ*kdnase* strains on minimal media supplemented with varying carbon sources. Wild type and Δ*kdnase* strains were plated on solid Kafer’s minimal medium (fungal media), supplemented with various carbon sources. Sialic acid is *N*-acetylneuraminic acid (Neu5Ac). Equivalent numbers of conidia were inoculated into the center of each plate and plates were incubated for 72 h at 37°C. Growth differences between the parental strain and the knockout are noted with boxes. Hygromycin resistance is the Δ*kdnase* selectable marker and the mutant but not the wild type grew in the presence of hygromycin, as expected. Three other Δ*kdnase* mutants displayed the same phenotype (data not shown).

A growth assay was performed to determine how the loss of the *kdnase* gene affected the nutritional requirements of *A. fumigatus*. Strains were grown for 72 h on Kafer’s minimal medium containing only the specified carbon source. When grown on minimal media supplemented with Kdn, glucose, or mannose, the growth of the Δ*kdn*ase strain was inhibited compared to WT (**Figure 1**). Compared to Kdn, Neu5Ac (labeled sialic acid in **Figure 1**) is a poor carbon source for *A. fumigatus*; however, the *Δkdnase* strain had even lower growth than WT on Neu5Ac. These data also confirm our previous finding that Kdn is strongly preferred to Neu5Ac in *A. fumigatus* (Telford et al., 2011).

### Sorbitol Inhibits the Growth of the *Δkdnase* Strain

**FIGURE 2:**
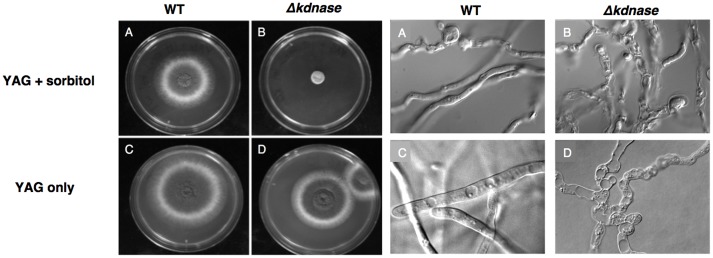
Growth of the Δ*kdnase* strain is inhibited compared to the wild type strain when grown on solid medium supplemented with 1 M sorbitol. **Left**: YAG plates (with or without 1 M sorbitol) were inoculated with 10^5^ conidia, in a volume of 5 μL in the center of each plate and incubated at 37°C for 48 h. **(A)** WT on YAG+sorbitol; **(B)** *Δkdnase* on YAG+sorbitol; **(C)** WT on YAG; **(D)** Δ*kdnase* on YAG. **Right:** Slide cultures were prepared for each strain on YAG ± sorbitol and cultures were grown for 20 h at 37°C. All DIC images were captured at 1000× magnification. The same letters correspond to the samples on the plate cultures shown on the **Left**.

We examined the relative growth and morphology of the WT and mutant strains in the presence of a hyperosmotic stress generated by addition of 1 M sorbitol to rich medium (YAG). Growth of the WT and *Δkdnase* strains was similar on solid YAG medium although the morphology of the KO strain was abnormal. However, the *Δkdnase* strain showed poor growth on YAG supplemented with sorbitol (**Figure 2**). A dose response experiment showed that growth inhibition was evident at concentrations of sorbitol of 0.4 M (data not shown). DIC images of slide cultures of the two strains showed that the morphology of the *Δkdnase* hyphae was abnormal even in YAG alone with hyperbranching and swollen filaments (**Figure 2** and Supplementary Figure S4). Quantitative analysis in RPMI-sorbitol medium showed that growth of the KO strain was <10% of WT after 48 h though some recovery was evident after 72 h (Supplementary Figure S4).

### Deletion of *kdnase* Alters *A. fumigatus* Ultrastructure and Causes Compensatory Cell Wall Thickening under Hyperosmotic Stress

**FIGURE 3:**
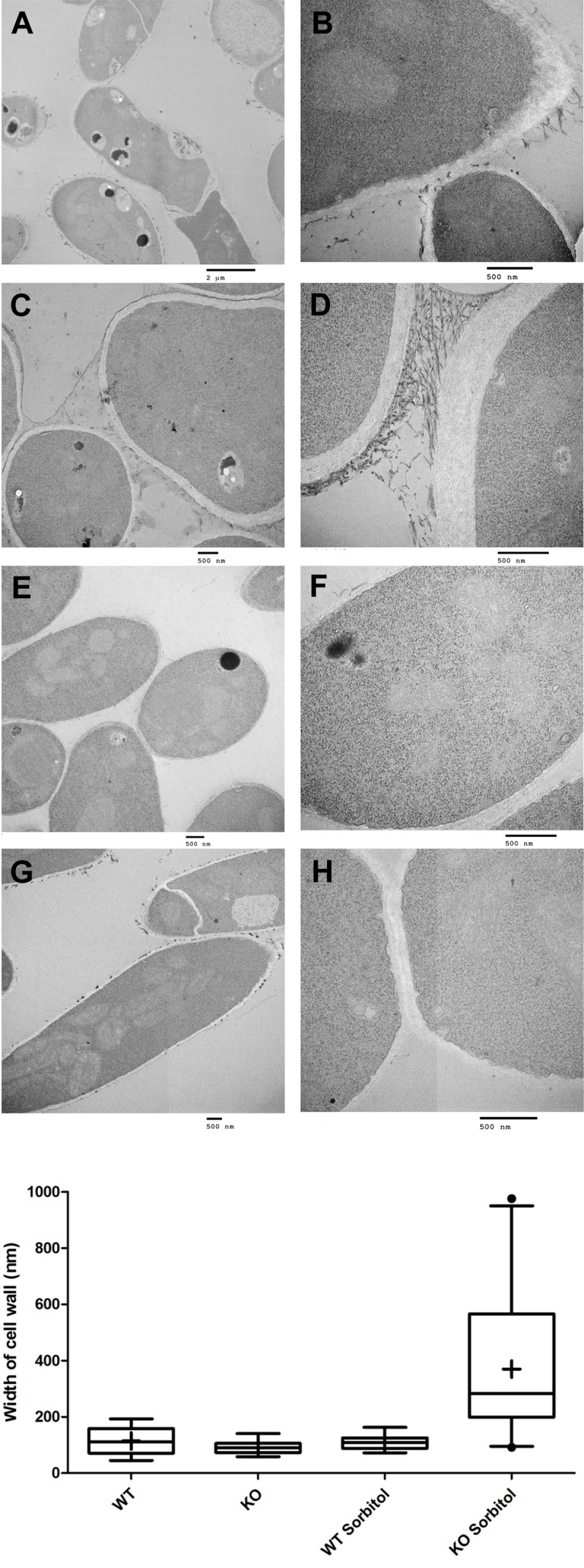
Transmission electron microscope images show that sorbitol increases cell wall thickness of the *Δkdnase* strain. **(A)** Each row represents one strain/growth condition at lower (Left) and higher (Right) magnification. **(A,B)** - *Δkdnase* knockout grown in YAG; **(C,D)** - *Δkdnase* knockout in YAG + sorbitol; **(E,F)** - WT in YAG; **(G,H)** - WT in YAG + sorbitol. Note the difference in cell wall thickness in **(D)** and **(H)**. Scale bar is below each image. **(B)** From the TEM images of hyphae, cell wall thickness was quantified with ImageJ using the measure function. Cell wall thickness was measured 5 times in each image at coordinates determined by random number generation. The number of separate images analyzed for each condition is as follows: WT – 17, Δ*kdnase* (KO) – 17, WT + 1M sorbitol – 12, Δ*kdnase* + 1M sorbitol – 24. Whiskers represent the 5–95% confidence interval, the dots indicate outliers, and the + indicates the median. The KO plus sorbitol group was significantly difference from all other groups at *p* < 0.001 (ANOVA followed by multiple comparison test with the Bonferroni correction).

We examined the surface structure of conidia and hyphae of the WT, *Δkdnase*, and *Δkdnase^R^* strains grown in RPMI using scanning electron microscopy. No apparent structural changes in conidia were evident (data not shown); however, *Δkdnase* hyphae had a less dense glycocalyx, a structure that is principally composed of polyanionic exo-polysaccharides. The rescued strain had an intermediate phenotype (Supplementary Figure S6). We quantified galactosaminogalactan (GAG) content of the three strains using lectin staining [soybean agglutinin (SBA) linked to a fluorophore] but found no significant differences (data not shown). Hence, changes in surface structure in the knockout cannot be attributed to alterations in GAG content. TEM images of hyphae showed that the cell walls of the *Δkdnase* hyphae (panels **A–D** in **Figure 3A**) appeared thicker than those of WT (panels **E–H** in **Figure 3A**) when strains were grown in medium supplemented with sorbitol (compare panels **C,D** with panels **G,H**); this was confirmed by quantitative analysis of the images (**Figure 3B**). These data suggest that compensatory cell wall thickening occurred in response to the hyperosmotic stress in the *Δkdnase* strain but not the WT strain.

### The *Δkdnase* Strain Is Resistant the Growth Inhibitory Effects of Congo Red and Calcofluor White

**FIGURE 4:**
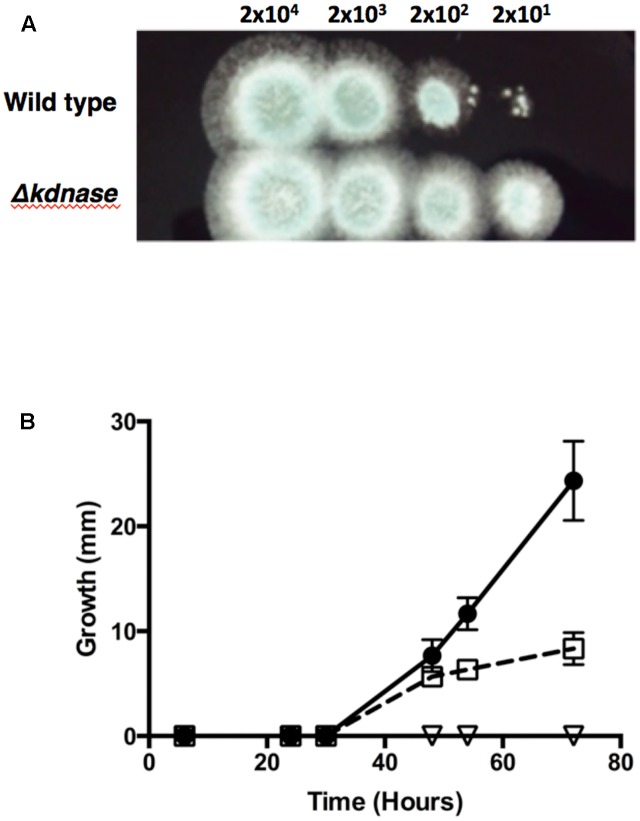
The Δ*kdnase* knockout strain is resistant to the growth inhibitory effects of Congo Red and Calcofluor White. **(A)** Wild type or Δ*kdnase* conidia were plated in a series of 10-fold dilutions in a total volume of 2 μl on YAG containing 500 μg/mL of Congo Red. **(B)** Conidia (∼5000) from each strain were plated on solid YAG media containing 0.25% Calcofluor White and colony diameter was measured after growth at 37°C. 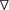 - wild type; 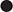 - Δ*kdnase* knockout strain; and 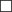 - Δ*kdnase^R^* strain. Values represent the mean ± SD of three experiments.

Chitin is an important structural polysaccharide of the *A. fumigatus* cell wall (Lee and Sheppard, 2016) and its synthesis is upregulated under conditions of cell wall stress. The dyes Congo Red (CR) and Calcofluor White (CW) were used to probe changes in the cell wall chitin content as these dyes bind structural polysaccharides with a preference for chitin (Roncero and Duran, 1985). **Figure 4A** and Supplementary Figure S5 show that the *Δkdnase* strain was able to grow in concentrations of CR that almost completely inhibited WT growth on solid media. The rescued strain had an intermediate phenotype. The results with CW were similar: After 72 h of growth on YAG supplemented with 0.25% CW, the WT strain showed no growth whereas the *Δkdnase* strain reached ∼25 mm of radial growth. The *Δkdnase^R^* strain grew to only 30% of the level of the *kdnase* knockout strain (**Figure 4B**).

### Hyperosmotic Stress Increases Cell Wall α(1,3)-Glucan and Chitin Levels in the *Δkdnase* Strain

**FIGURE 5:**
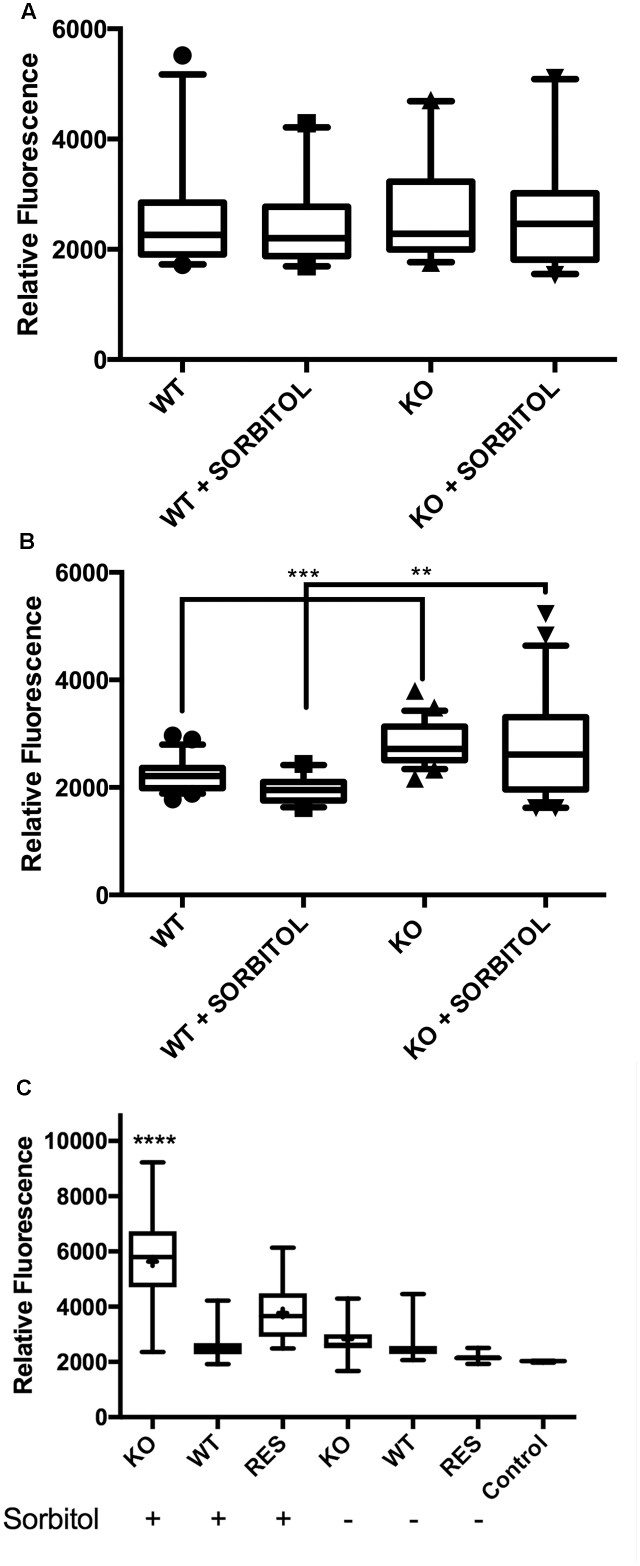
Hyperosmotic stress increases cell wall α(1,3)-glucan and chitin levels in the *kdnase* strain. Cell wall polysaccharides **(A)** β(1,3)-glucan; **(B)** α(1,3)-glucan; and **(C)** chitin were determined in hyphal tips grown in slide culture on YAG medium with or without sorbitol (1 M). Live cells were labeled with fluorescent antibodies **(A,B)** or the chitin probe **(C)**, imaged with confocal microscopy and the fluorescence yield was quantified with Volocity software. Representative images of chitin staining are shown in Supplementary Figure S6. WT, wild type; KO, Δ*kdnase* strain and Res, Δ*kdnase^R^* strain.

To directly quantify the levels of selected cell wall polysaccharides, strains were grown in slide culture on YAG medium with or without sorbitol (1 M) and immunofluorescence microscopy was used to measure the β(1,3)-glucan, α(1,3)-glucan and chitin contents of the hyphae. **Figure 5** shows that although no differences were detected in β(1,3)-glucan, α(1,3)-glucan reactivity was significantly increased in *Δkdnase* hyphae in the presence or absence of sorbitol by approximately 30% (**Figures 5A,B**). Binding of the chitin probe was dramatically different in the three strains; the *Δkdnase* strain grown in sorbitol had a threefold higher level of fluorescence compared to all other strains and conditions (**Figure 5C** and Supplementary Figure S7). These data suggest that resistance to CR and CW and the observed cell wall thickening in hyperosmotic conditions may be a consequence of increased chitin deposition in the cell wall of the *Δkdnase* strain. Alternatively, the increased fluorescence may reflect increased access of the probe to chitin as a consequence of cell wall disorganization in the *Δkdnase* strain.

### Susceptibility to Various Antifungal Agents

**FIGURE 6:**
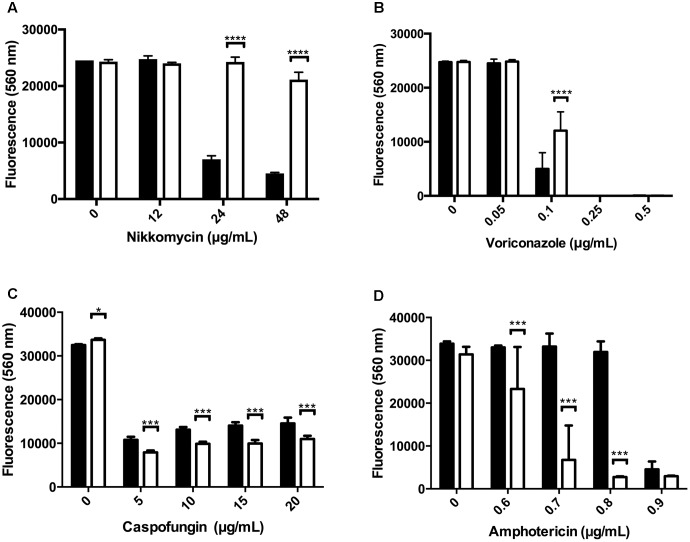
Susceptibility of WT and *kdnase* strains to various antifungal agents. Wild type and Δ*kdnase* conidia (2 × 10^5^ conidia/mL) were inoculated into 96-well microdilution plates containing RPMI supplemented with resazurin with increasing concentrations of one of the following antifungal agents: **(A)** nikkomycin, **(B)** voriconazole, **(C)** caspofungin and **(D)** amphotericin B. Plates were incubated at 37°C for up to 46 h. Growth was quantified by fluorescence (excitation = 560 nm; emission = 590 nm). Control wells contained no antibiotic or no conidia. Error bars indicate the 95% confidence interval of 6 replicates per condition. Significant differences in growth between the wild type and Δ*kdnase* strains are indicated by ^∗∗∗∗^(*p* < 0.0001), ^∗∗∗^(*p* < 0.001) or ^∗^(*p* < 0.05). Each antibiotic was tested at least 2 times with similar results. Black bars - wild type; white bars - Δ*kdnase*.

We next tested the relative susceptibility to several antifungal agents. Nikkomycin Z is a competitive inhibitor of chitin synthase (Hector, 1993). The *Δkdnase* strain was more resistant to nikkomycin than WT (**Figure 6A**) even though WT *A. fumigatus* strains already have high minimum inhibitory concentration values for nikkomycin (16–64 μg/mL) that limit its clinical use (Ganesan et al., 2004). The *Δkdnase* strain also showed some resistance to voriconazole, a drug that alters membrane sterol composition by inhibiting cytochrome P450 lanosterol demethylase (Odds et al., 2003), but resistance was only detected in a narrow voriconazole concentration (**Figure 6B**). We did not detect a difference in the susceptibility of the *Δkdnase* strain to itraconazole, another triazole antifungal (data not shown). In contrast, *Δkdnase* was more susceptible than WT to both caspofungin and amphotericin B (**Figures 6C,D**). Caspofungin is an echinocandin antifungal that inhibits the Fks1 subunit of β(1,3)-glucan synthase (Odds et al., 2003). It is the only anti-fungal agent that directly targets the cell wall. At caspofungin concentrations <1 μg/mL, no difference in growth was observed between the two strains (data not shown); however, at >5 μg/mL caspofungin (concentrations at which ‘paradoxical’ growth occurs), *Δkdnase* growth was marginally, but significantly, reduced compared to the WT strain (**Figure 6**). Finally, *Δkdnase* also showed increased sensitivity to Amphotericin B (AB) compared to WT (∼ 6-fold at 0.7–0.8 μg/mL) (**Figure 6**). AB is a pore-forming polyene antibiotic used as an alternative to voriconazole in primary treatment of IA. Because of the clinical importance of AB and the *in vitro* sensitivity of the *Δkdnase* knockout strain, we included AB treatment in our test of *AfΔkdnase* virulence.

### The *Δkdnase* Strain Has Reduced Virulence and Fungal Burden in Combination with Amphotericin B Treatment in a Neutropenic Mouse Model of Invasive Aspergillosis

**FIGURE 7:**
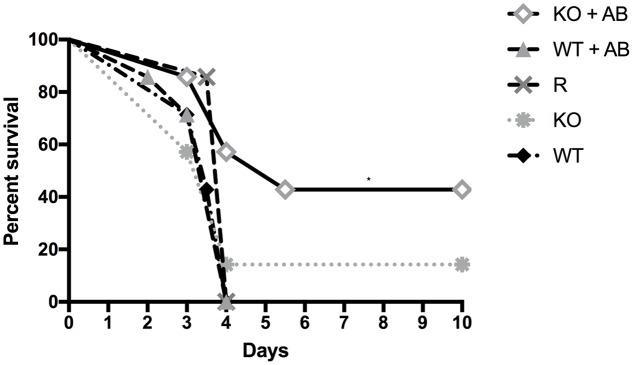
Kaplan-Meier survival curves of immunosuppressed mice exposed to wild type (WT), *Δkdnase* (KO), or *Δkdnase^R^* (R) conidia with or without AmBisome (AB) treatment. All mice were immunocompromised with cyclophosphamide and cortisone. On day 0, mice in all treatment groups (7 per group) received 10^6^ conidia intranasally of the appropriate strain, and indicated groups were also treated with i.v. AB (5 mg/kg), 4 h after exposure to conidia. Control mice that received saline intranasally (3) or saline intranasally followed by i.v. AB (4) had 100% survival (not shown). Comparison of survival curves with the log rank test (Mantel-Cox) was significant at *p* < 0.0007. ^∗^Indicates *p* < 0.04 for KO+AB versus WT+AB.

**FIGURE 8:**
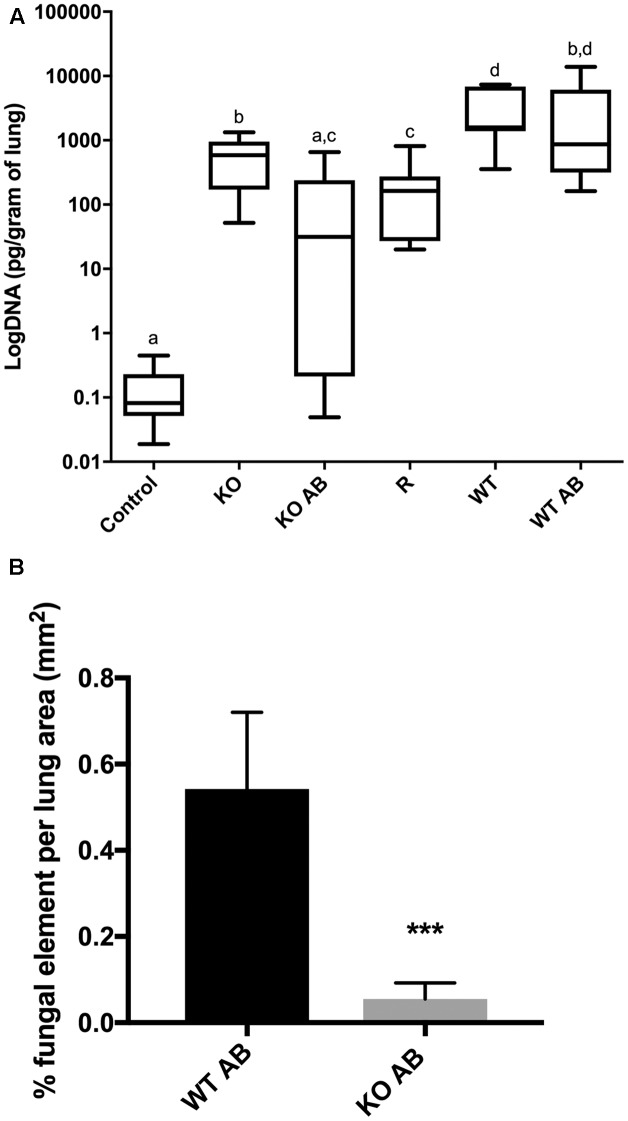
Lung fungal burden. **(A)** DNA was extracted from homogenized lung samples (*n* = 7 except KO+AB was *n* = 6) and fungal DNA was quantified by TaqMan qPCR. Fungal DNA content was determined by comparison to a standard curve and normalized to lung wet weight. Statistical analysis was carried out using a one-way ANOVA followed by a Games Howell test for differences between groups. Groups with the same letters are not significantly different at *p* < 0.05. Control animals received saline only and represent the pooled values from animals with and without AB treatment. **(B)** Percent coverage of GMS stained lung sections by fungal elements (conidia or hyphae). Mean percent fungal element was quantified from 3 complete lung sections from 7 WT AB mice and 6 KO AB mice taken at endpoint using the color threshold tool in ImageJ; error bars represent the S.E.M. ^∗∗∗^ KO AB was significantly different from WT AB at *p* < 0.0009 using ANOVA followed by Tukey’s test on log-transformed data. All CON AB values were 0 (not shown). Abbreviations are the same as in the legend to **Figure 7**. Representative images of GMS-stained lung sections are shown in **Figure 9**.

To test the virulence of the *Δkdnase* knockout strain and the efficacy of AB treatment *in vivo*, female BALB/cJ mice were first immunosuppressed with cortisone and cyclophosphamide as described in the Section “Materials and Methods.” On day 0, mice in all treatment groups (seven per group) received 10^6^ conidia intranasally of the appropriate strain. Two groups of animals were also treated with AmBisome (AB) (5 mg/kg i.v.) 4 h after exposure to *Δkdnase* (KO), or *Δkdnase^R^* (R) conidia. Two additional groups of control mice received the same immunosuppressive regime and were exposed to saline intranasally (*n* = 3) or saline followed by AB treatment (*n* = 4). Control mice (not infected with *A. fumigatus)* had 100% survival (data not shown). Kaplan-Meier survival curves of immunosuppressed mice exposed to wild type (WT), *Δkdnase* (KO), or *Δkdnase^R^* (R) conidia with or without AmBisome treatment are shown in **Figure 7**. All mice were weighed and monitored by a blinded observer twice per day for up to 10 days (experimental endpoint): Mice showing signs of morbidity approaching a humane endpoint were sacrificed. Most mice reached a humane endpoint by day 4 with the exception of one infected with the *Δkdnase* strain (KO) (1/7 survived) and four mice infected with the *Δkdnase* strain that also received AmBisome treatment following spore exposure (KO+AB) (3/7 survived for 10 days). These surviving mice also gained weight and had no apparent symptoms (data not shown). Using the log rank test, a significant difference in survival was found (*p* < 0.0007). Comparison of KO+AB and WT+AB were significantly different at *p* < 0.04. Fungal burden at endpoint was assessed by two methods. Fungal DNA content was measured by qPCR and data were analyzed using a one-way ANOVA followed by a Games Howell test for differences between groups. The *Δkdnase* plus AmBisome (KO+AB) cohort was the only group in which fungal burden was not statistically different from the controls (CON+AB), and the median fungal burden in the KO+AB treatment group was 10-fold lower than in WT+AB (**Figure 8A**). We also quantified fungal elements in 3 lung sections from each animal in the WT+AB (*n* = 7) and KO+AB (*n* = 6) treatment groups and the data are summarized in **Figure 8B**. The percent coverage of the lung by fungal elements in GMS-stained lung sections corroborated the fungal DNA burden data: WT+AB mice had values ∼10-fold higher than the KO+AB group. Thus, the increased susceptibility of the *Δkdnase* strain to amphotericin B we observed *in vitro* (**Figure 6**) was replicated *in vivo* and resulted in a lower fungal burden and greater survival of the host in the KO+AB group.

Exposure to the *Δkdnase* strain resulted in a significantly lower lung fungal burden compared to wild type with or without AmBisome treatment, even in the animals that died from the disease. To determine whether the relative growth of the two strains may have contributed to this difference, we measured growth in minimal essential medium (MEM) supplemented with 15% human serum. After 18 and 25 h at 37°C, the *Δkdnase* strain had approximately 50% of the growth of the WT strain but by 48 h, cell density was equivalent between the two strains (data not shown). Moreover, in the antibiotic sensitivity experiments after 28–48 h, the growth of WT and *Δkdnase* strains were equivalent in the no antibiotic control samples (**Figure 6**). Finally, the data in **Figure 7** show that mice exposed to the *Δkdnase* strain without AB treatment did not survive longer than mice that were given the WT strain. We confirmed this in a separate mouse study in which we found that the survival of BALB/c female mice was the same in the WT or *Δkdnase* treatment groups (data not shown). This suggests the combination of amphotericin B with *Δkdnase* deletion was responsible for the lower fungal burden and increased survival.

**FIGURE 9:**
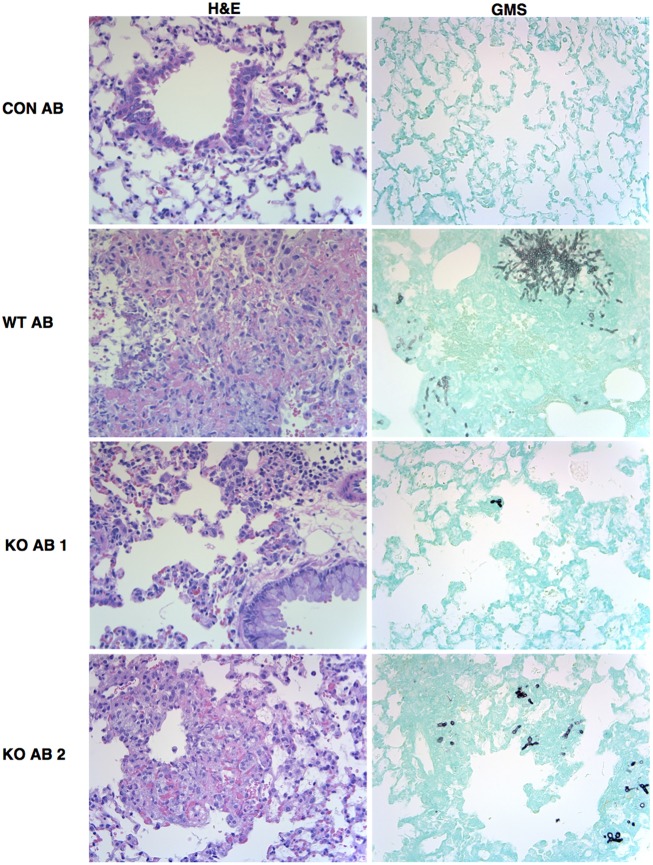
Representative images of lung histology. Fixed lung sections were stained with hematoxylin and eosin (H&E) to visualize cell infiltrate or Grocott’s methenamine silver (GMS) to stain fungal elements (fungi appear black against the green host tissue). All images were obtained at 400× magnifications. Abbreviations are the same as described in the legend to **Figure 7**. Images from lung sections taken from two different mice in the KO+AB group are shown.

Representative sections of H&E and GMS stained lung sections from AmBisome-treated animals are shown in **Figure 9**. Extensive cellular infiltrates and hemorrhage were evident in WT+AB group, and clusters of fungal filaments were abundant in lung sections. In the KO+AB mice, although inflammation and fungal elements were evident, these were more restricted in size and less frequently observed (**Figure 9**). GMS-stained sections from mice exposed to WT, *Δkdnase*, or *Δkdnase^R^* without AB treatment are shown in Supplementary Figure S8.

### Leukocytes in Lung Sections

**FIGURE 10:**
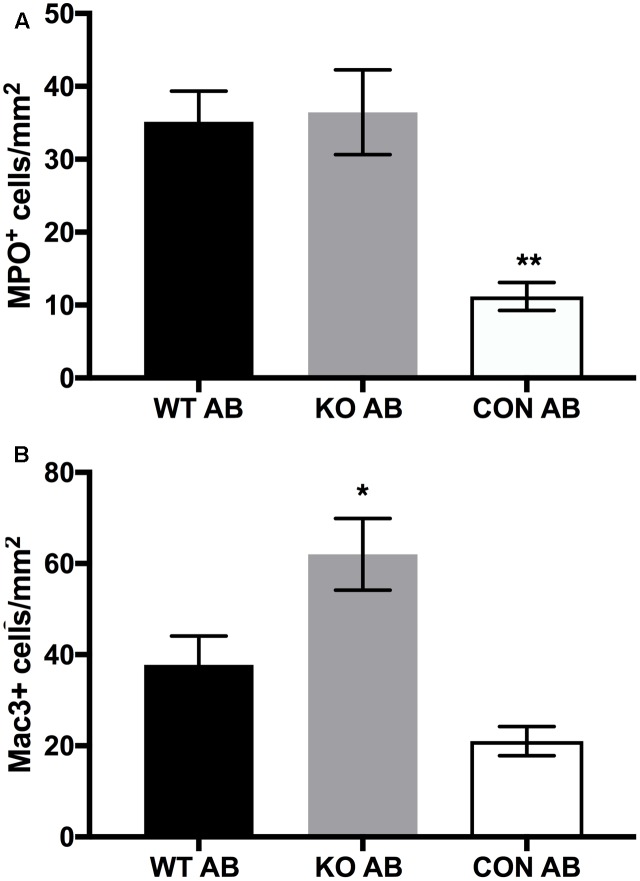
Leukocyte counts in lung sections. Whole lung sections (3 per animal) were fixed and stained to detect neutrophils (MPO^+^) cells **(A)**, or macrophages (Mac3+) **(B)**. Data represent the mean of *n* = 7 (WT AB), *n* = 6 (KO AB) and *n* = 3 (CON AB) ± SEM. **(A)** MPO^+^ cells/mm^2^ were not significantly different in lungs of WT AB and KO AB animals; ^∗∗^CON AB values were significantly lower than WT AB and KO AB at *p* < 0.01 (ANOVA followed by Tukey’s multiple comparison test). **(B)** The number of Mac3+ cells was significantly higher in KO AB samples compared to CON AB ^∗^(*p* < 0.05) whereas Mac3+ values for WT AB were not significantly different from KO AB or CON AB (one-way ANOVA followed by Tukey’s multiple comparisons test). Abbreviations are the same as in the legend of **Figure 7**. Sample images of Mac3+ staining are shown in Supplementary Figure S9.

We examined the relative numbers of macrophages and neutrophils in lung sections from the AmBisome treatment groups at endpoint using immunohistochemistry. Neutrophil (MPO-positive cells) counts/mm^2^ in the lung of animals that received either WT or *Δkdnase* conidia were elevated threefold over controls (**Figure 10A**); no significant difference was observed between the treatment groups. Macrophages (Mac 3-positive cells) were higher than control animals for both groups receiving *A. fumigatus*; however, the accumulation of macrophages detected in tissues of mice exposed to WT conidia was significantly lower than in *Δkdnase*-exposed mice (**Figure 10B**). Supplementary Figure S9 contains representative photomicrographs showing the difference in the number and staining of the macrophages in a lung section from a mouse in the KO+AB group compared to one from WT+AB.

### Uptake of Conidia by Cultured Murine Macrophages

**FIGURE 11:**
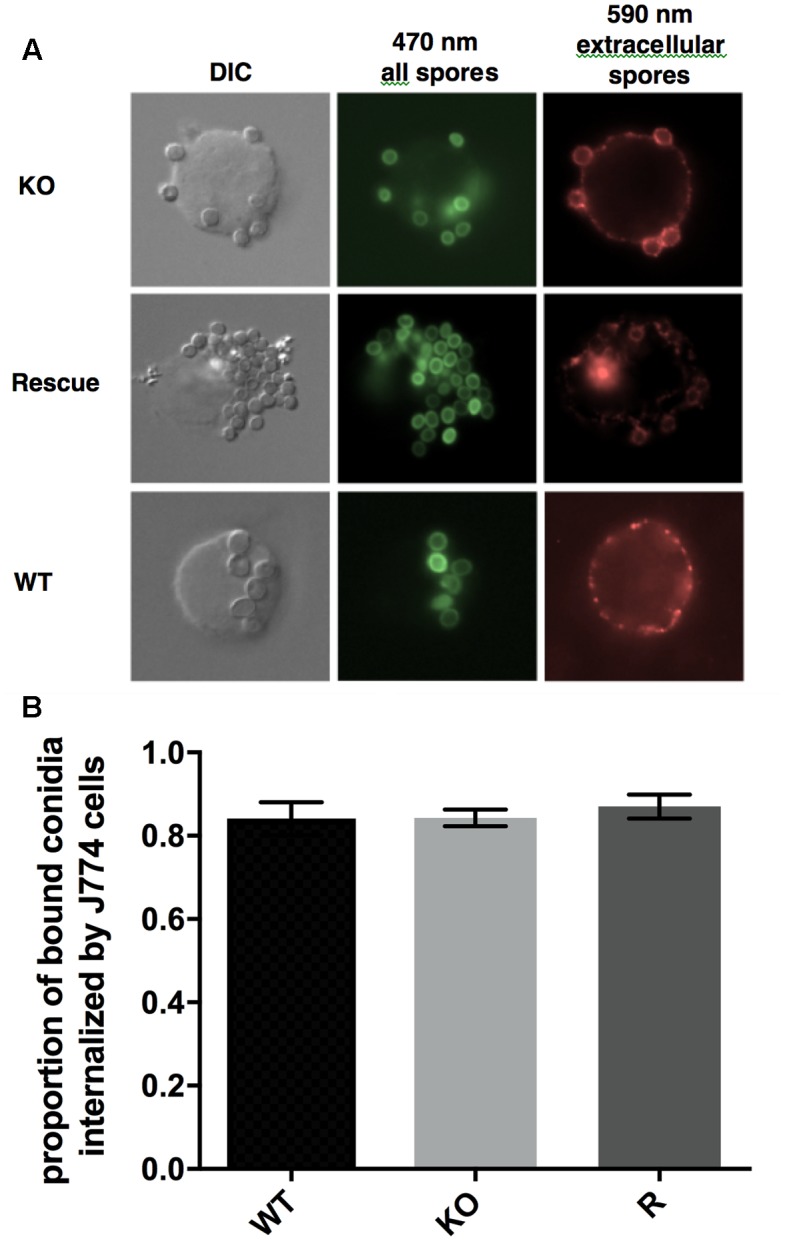
Uptake of conidia by cultured murine macrophages. **(A)** Microscopic analysis of the uptake of the fungal conidia by J774 macrophages after 1 h of co-incubation at 37°C. Green fluorescence from FITC shows all conidia (470 nm) whereas only external conidia were stained by the antibody (red – 590 nm). **(B)** Proportion of bound conidia internalized by cultured macrophages was equivalent in all three strains. Data show the mean ± SEM from a minimum of 28 images per strain. The experiment was repeated twice with the same result. Abbreviations are the same as those described in the legend **Figure 7**.

Uptake and killing of conidia by macrophages is an important innate immune defense against *A. fumigatus* (Philippe et al., 2003). We compared the uptake of FITC-labeled WT, *Δkdnase*, and *Δkdnase^R^* conidia, quantified with immunofluorescence microscopy in the cultured mouse macrophage cell line, J774. No difference in uptake by J774 cells was observed between the 3 strains (**Figure 11**) and a similar experiment that measured conidial uptake by cultured human type 2 pneumocytes (A549 cells) produced the same result (data not shown). Thus, *Δkdnase* conidia were internalized to the same extent as WT by both professional phagocytes and epithelial cells; however, whether there were differences in the rate of conidial killing was not determined.

## Discussion

Our study revealed an important role for the *Aspergillus fumigatus* sialidase (Kdnase) in maintaining hyphal cell wall stability in the presence of stressors such as hyperosmotic stress or selected antimicrobial agents. Deletion of *kdnase* increased the survival of mice that were treated with amphotericin B. To our knowledge, this is the first report that implicates a sialidase in cell wall homeostasis and virulence in fungi.

The *Aspergillus fumigatus* hyphal cell wall has an inner wall containing β(1,3)-glucans, chitin, galactomannan (linked to chitin), galactosaminogalactan, and an outer cell wall composed of α(1,3)-glucans, galactomannan [covalently linked to β(1,3)-glucan] covered in a loose sheath of galactosaminogalactan (Lee and Sheppard, 2016). Aberrant hyphal morphology and excess chitin deposition were detected in the *Δkdnase* knockout strain in response to cell wall stressors whereas β(1,3)-glucan levels were unchanged. Upregulation of chitin synthesis in response to cell wall stress is a well-known phenomenon that involves the cell wall integrity (CWI) pathway suggesting that deletion of *kdnase* weakened the fungal cell wall. The CWI sensor proteins (WSC) are plasma membrane receptors that detect mechanical stress (Grice et al., 2013; Valiante et al., 2015) and are connected to the downstream mitogen-activate protein kinase (MpkA) cascade by the protein kinase C (PkcA). An *A fumigatus* strain with a single amino acid substitution in PkcA (G579R), causing reduced Mpk-activating function, showed an increased sensitivity to CW, CR, and SDS, indicative of cell wall dysfunction (Rocha et al., 2015). The CWI pathway and the massive redundancy in some cell wall genes [e.g., *A. fumigatus* contains 8 chitin synthase genes (Muszkieta et al., 2014)] make the cell wall a difficult therapeutic target; however, the targeting difficulty is outweighed by the cell wall’s importance for fungal survival and the fact that the host lacks a cell wall.

Growth of the *Δkdnase* strain was inhibited by hyperosmotic stress. In fungi, activation of the high osmolarity glycerol (HOG) pathway permits growth in hyperosmotic conditions, for example, by increasing expression of genes involved in glycerol biosynthesis (Bahn, 2008). Cross-talk between the CWI and the pathways is well established in *S. cerevisiae* (Fuchs and Mylonakis, 2009) and there is evidence from transcriptomic studies that they are also linked in *A. fumigatus* (Malavazi et al., 2009). The Type III hybrid histidine kinase, TscC both senses and signals downstream adaptation to prevent osmotic stress. The *A. fumigatus ΔtscC* mutant had a phenotype very similar to our *AfΔkdnase* mutant including growth inhibition by sorbitol and resistance to Congo Red dye (McCormick et al., 2012). Interestingly, pharmacological activation of the HOG pathway in *A. fumigatus* with fludioxonil resulted in growth arrest and a remodeled hyphal cell wall, including increased deposition of chitin (Wiedemann et al., 2016). Further studies are required to determine whether the HOG pathway genes or gene products are altered in *Δkdnase*.

We were able to obtain only a partial rescue phenotype *ΔkdnaseR* despite attempting several strategies including ectopic insertion of *kdnase* with native promoter, ectopic insertion of *kdnase* with constitutive promoter, and homologous re-insertion of the gene at the correct locus. We also made a rescue construct containing the recently annotated 3′ untranslated region (UTR); 3′ UTR regions are known to regulate the translation, location and stability of mRNA transcripts (Mignone et al., 2002; Zhang and Sachs, 2015). However, no improvement of the rescued phenotype was observed (data not shown). Alternatively, epigenetics, or the environment surrounding the *kdnase* locus, could be responsible for the difficulty in achieving full complementation. Difficulty in producing a full rescue strain for a sialidase knockout mutant in *M. tuberculosis* was reported by May et al. (2012).

*AfΔkdnase* was more sensitive than WT to caspofungin *in vitro*. Caspofungin is a lipo-peptide that inhibits β(1,3)-glucan synthase resulting in growth arrest in *A. fumigatus*. However, at higher drug concentrations (>1 μg/mL), caspofungin stimulates the growth of *A. fumigatus*, a phenomenon called the ‘paradoxical effect’ (Wiederhold, 2007). Fortwendel et al. (2010) showed that paradoxical growth was related to up-regulation of chitin synthesis via calcineurin signaling. In the *Δkdnase* strain as with WT, growth increased at higher caspofungin concentrations, likely secondary to chitin synthesis, but growth of *Δkdnase* was less than WT (Fortwendel et al., 2010). This suggests that, even with compensatory chitin deposition, the *Δkdnase* strain was more susceptible to the effects of caspofungin on cell wall structure.

The *Δkdnase* strain was also more sensitive to amphotericin B (AB) *in vitro* and *in vivo*. AB is a polyene anti-fungal agent and the first anti-fungal used clinically to treat IA (Mesa-Arango et al., 2012). Its mechanism of action is still unclear but involves pore formation in the plasma membrane upon hydrophobic interaction with ergosterol, the fungal membrane sterol (Urbina et al., 1987), as well as sterol sequestration (Palacios et al., 2011; Gray et al., 2012) and induction of oxidative stress (Sangalli-Leite et al., 2011; Kim et al., 2012). Various studies have found a relationship between fungal cell wall patency and AB susceptibility. For example, the relative susceptibility of the *Kluyveromyces, Candida* and *Schizosaccharomyces* spp. to AB was correlated with cell wall chitin content but not to plasma membrane ergosterol content. Species or strains with high levels of chitin in their cell wall were shown to be more susceptible to challenge with amphotericin B (Bahmed et al., 2003). In the yeast pathogen, *Cryptococcus neoformans*, Banerjee et al. (2016) created a *ura1*Δ mutant lacking dihydroorotate dehydrogenase that showed both compromised cell wall structure and increased susceptibility to AB. They proposed that decreased nucleotide-sugar pools in the mutant resulted in structural defects in the cell wall that in turn, allowed a more rapid entry and greater binding of AB to the plasma membrane. Thus, the remodeling of cell wall structure that occurs during stress or in certain mutant strains such as *Δkdnase* can lead to increased AB susceptibility.

The number of macrophages was significantly higher in KO+AB mice and this was positively correlated with survival (*R*^2^ = 0.73). Alveolar monocyte recruitment into the mouse lung during inflammation is dependent on CCR2 (Maus et al., 2003) which is the receptor for the chemokine CCL2. Neutrophil and monocyte recruitment into the lung are interdependent so as to provide a coordinated inflammatory response during infection (Maus et al., 2003).

*In vitro*, the proportion of *Δkdnase* and WT conidia internalized by J774 mouse macrophages was equivalent. This indicates that the fungal-specific pathogen-associated molecular patterns (PAMPs) in the *Δkdnase* strain were recognized by macrophage pattern recognition receptors (PRRs). The C-type lectin receptors Dectin-1 and Dectin-2, and DC-SIGN bind fungal β(1,3)-glucans, α-mannans and galactomannans, respectively (Erwig and Gow, 2016). Although we did not measure the galactomannan and mannan contents of the cell wall, β(1,3)-glucan levels were unchanged in the *Δkdnase* strain which may explain why we found no change in internalization. Many investigators have demonstrated that a small proportion of *A. fumigatus* conidia may germinate within the phagosome, effectively escaping from the cell (Schaffner et al., 1982; Wasylnka and Moore, 2003). Immune suppression by cortisone directly reduces the effectiveness of macrophage-mediated killing of *A. fumigatus*, allowing a greater proportion to escape (Schaffner et al., 1982). We hypothesize that the lower numbers of macrophages in the WT+AB mice may have been a consequence of macrophage destruction by germinating WT conidia although this needs to be confirmed experimentally.

To date, Kdn has been identified in relatively few organisms/tissues though its presence is likely underreported. It has been isolated from amphibian egg jelly coats (Strecker et al., 1992; Inoue and Kitajima, 2006), fish skin and reproductive tissues (Nadano et al., 1986; Kanamori et al., 1989; Song et al., 1991) and some bacteria (Shashkov et al., 2002; Ostash et al., 2014). In humans, only traces of Kdn were detected on glycoproteins and glycolipids present in the erythrocyte plasma membrane (Bulai et al., 2003). Interestingly, Kdn has also been detected in human cancer tissue (Inoue et al., 1996, 1998). Preliminary evidence from our laboratory suggests that *A. fumigatus* may also contain Kdn (Zandberg et al., Unpublished results). Further study is required to precisely define the distribution and level of Kdn in the WT and *Δkdnase* strains, and to determine whether Kdn is found in other fungi.

There is a large body of literature that clearly demonstrates the importance of sialidases in microbial virulence, particularly in bacteria. Much less is known about Kdnases and to our knowledge, other than in *A. fumigatus*, no other *kdnase* knockout strains have been constructed. The bacterium, *Sphingobacterium multivorum* has a Kdn-specific sialidase that, unlike *Af* Kdnase, is incapable of using Neu5Ac as a substrate (Nishino et al., 1996). Predicted sialidase enzymes are present in other pathogenic *Aspergillus* species including *A. terreus*, *A. clavatus, A. sydowii*, and *A. lentulus* and in several dermatophytes; however, whether these are Kdnases has not been established. No Kdnase enzyme has been identified in any mammal. Humans express four sialidase isozymes, NEU1–NEU4, that differ in substrate specificity and tissue localization (Smutova et al., 2014). All human sialidases are inhibited by the sialidase transition state analog DANA (Neu5Ac2en) with IC_50_ values that range from 6 to 90 μM (Albohy et al., 2013). In contrast, we previously showed that Neu5Ac2en did not inhibit the recombinant *A. fumigatus* Kdnase even at concentrations up to 10 mM, but was inhibited by the related Kdn analog, 2,3-didehydro-2,3-dideoxy-D-*glycero*-D-*galacto*-non-2-ulosonic acid (Kdn2en). Furthermore, Kdn2en bound to the enzyme in a 1.84 Å-resolution crystal structure (Telford et al., 2011). Thus, it is possible to selectively inhibit Kdnase without affecting host sialidases. Because humans do not express a Kdnase, a specific inhibitor of this enzyme would be expected to have a good safety profile. Small-molecule inhibitors of viral sialidases are already in clinical use including the two important anti-influenza drugs, zanamivir and oseltamivir (von Itzstein, 2007). Thus, inhibition of *Aspergillus fumigatus* Kdnase might have therapeutic potential for the treatment of IA, especially when combined with amphotericin B. Such a combination treatment strategy may allow for reduced amphotericin B dose and accompanying toxicity. For this strategy to be successful, a small molecule Kdnase inhibitor must recapitulate the phenotype of the *kdnase* knockout.

## Author Contributions

Conceptualization: JN, ES, JY, and MM. Funding acquisition: MM and AB. Investigation: JN, ES, CS, AC, SM, JY, MH, and MM. Resources: MM, AB, JC, and KM. Visualization: JN, ES, CS, and MM. Writing – original draft: JN, ES, MH, AC, and MM. Writing – review and editing: JN, ES, JY, CS, AC, SM, AB, KM, JC, MH, and MM.

## Conflict of Interest Statement

The authors declare that the research was conducted in the absence of any commercial or financial relationships that could be construed as a potential conflict of interest.
