# Supplementary material for: The Aspergillus fumigatus Sialidase (Kdnase) Contributes to Cell Wall Integrity and Virulence in Amphotericin B-Treated Mice
Source: Front Microbiol. 2018 Jan 18;8:2706. doi: 10.3389/fmicb.2017.02706 (PMC5778107; doi:10.3389/fmicb.2017.02706)
Supplement: Supplementary file 1 [file Presentation_1.pdf]

Supplementary Material

The *Aspergillus fumigatus* sialidase (Kdnase) contributes to cell wall integrity and virulence in amphotericin B-treated mice

Jason R. Nesbitt[1¶], Elizabeth Y. Steves[1¶], Cole R. Schonhofer, Alissa Cait, Sukhbir S. Manku, Juliana H. F. Yeung, Andrew J. Bennet, Kelly M. McNagny, Jonathan C. Choy, Michael R. Hughes, Margo M. Moore*

*** Correspondence:** Margo Moore: mmoore@sfu.ca

1 Supplementary Figures

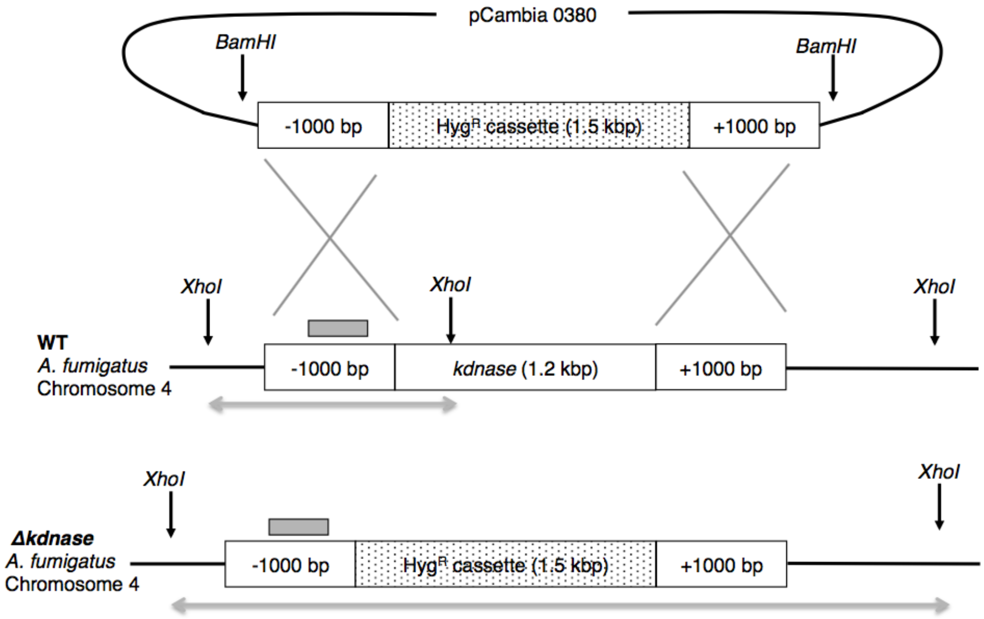

Figure S1. Schematic of *kdnase* gene knockout strategy and Southern blot analysis. The knockout construct (top) in pCambia contained the hygromycin resistance cassette (Hyg^R) (1.5 kbp: containing the *hph* gene from *E. coli* flanked by the *trpC* promoter and terminator sequences from *A. nidulans*) that was flanked by 1 kbp *kdnase* up- and downstream sequences. Homologous recombination via *Agrobacterium*-mediated transformation at *A. fumigatus* chromosome 4 at the *kdnase* coding region replaced the *kdnase* gene with the Hyg^R cassette. The location of the DIG-labelled probe used for Southern blotting is shown as a grey box. Double-sided grey arrows illustrate the size of the DNA segments that were probed after *XhoI* restriction digest of the DNA: for the wild

type locus, the expected size of segment binding to probe is 1595 bp, and in the *Δkdnase* strain, the expected size of segment is 4476 bp.

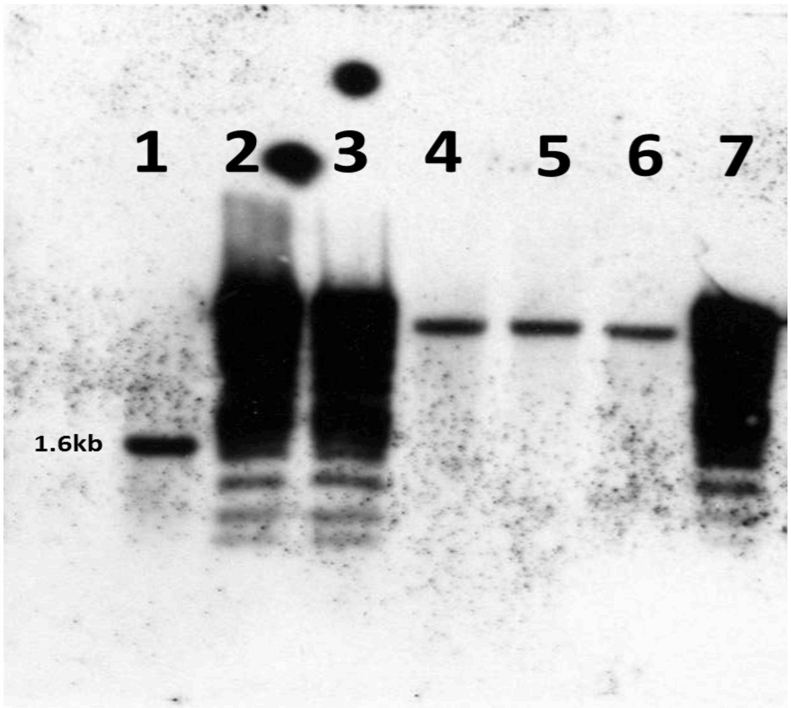

Figure S2. Southern blot confirming single copy homologous insertion of the knockout construct at the *kdnase* locus. Genomic DNA from wild type *A. fumigatus* and 3 putative *kdn*ase knockout mutants was digested with the restriction enzyme *Xho1* which cuts at a recognition sequence in the *kdn*ase gene and in the downstream region but not in the *hph* cassette (Fig S1). Genomic DNA to be used in the Southern blot was extracted via the CTAB method (Carlson et al. 1991). The probe was amplified from the downstream sialidase flanking region (242 bp downstream of the stop codon) by the primers 5'-TCACTGGGCTAATCCCTGAC and 5'-TGCACTCTCACTCCAAATGC. The probe was labeled using the DIG High-Prime DNA Labelling and Detection Starter Kit II (Roche) according to the manufacturer's protocol. PCR amplification of the probe was performed with iProof (Bio-rad) polymerase as per manufacturer's protocol. Lane 1 – wild type *A. fumigatus*; lanes 2,3 and 7 – size ladder; lanes 4-6 –*kdnase* knockout strains.

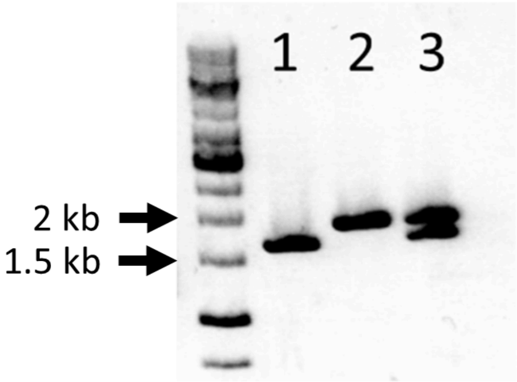

Figure S3. PCR screening for successful ectopic insertion of the wild type *kdnase* gene into the *Δkdnase* genome. PCR was performed on genomic DNA extracted from wild type, Δ*kdnase,* and putative rescued strains. Primers bound on either side of the Kdnase coding region: primer sequences were 5'-CGGAATCGGGAAGAAGTATT-3' and 5'-TCAGGATGTGATTTCACACGA-3'. Lane 1: WT - a band at 1.6 kb resulted from 400 bp of flanking sequence in addition to the 1.2kb *kdnase* gene; Lane 2: *Δkdnase* knockout strain- a band at 1.9 kb resulted from 400 bp of flanking sequence plus the 1.5 kb *hph* cassette. Lane 3: Rescued strain with ectopic copy of the *kdnase* gene - both bands amplified.

A.

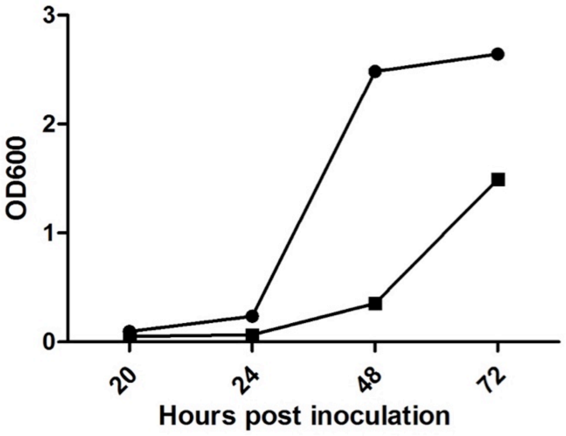

B.

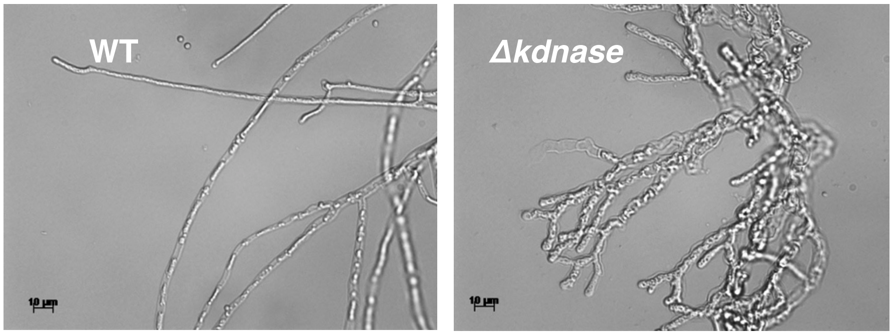

Figure S4. A. Growth of the Δ*kdnase* strain is inhibited by 1M sorbitol over 72 h of growth at 37 °C. Wild type and Δ*kdnase* conidia were inoculated into 96 well microdilution plates, containing RPMI plus sorbitol media supplemented with resazurin, at a concentration of $2x10^5$ conidia/mL and incubated at 37 ºC. Fluorescence from resorufin was monitored as an indicator of fungal growth (excitation wavelength = 560nm; emission wavelength = 590nm). Wild type - black circles; black squares - *Δkdnase*. **B. Morphology of WT and *Δkdnase* strains in slide culture after growth at 37°C for 24 h in YAG supplemented with 1M sorbitol.** Compared to WT, the *Δkdnase* hyphae have a hyperbranched phenotype.

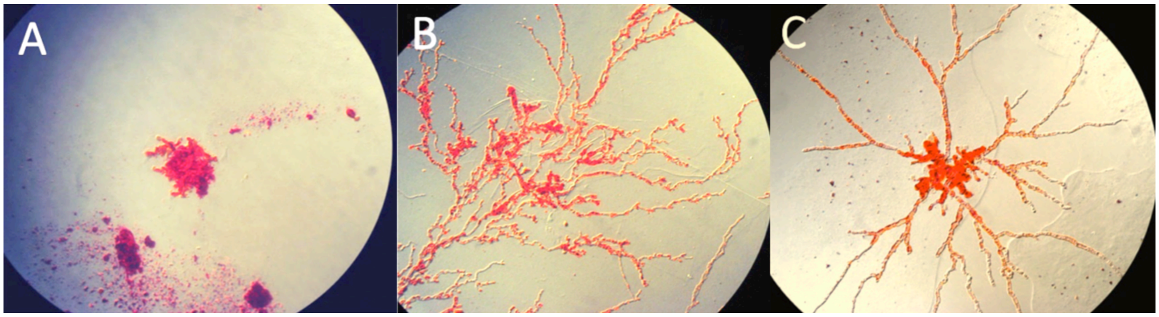

Fig S5. The phenotype of WT, *Δkdnase* and rescued strains in the presence of Congo Red. Slide cultures were prepared for each strain on YAG containing 750 µg/mL Congo Red dye and incubated for 20 h at 37°C. All images were obtained on a Zeiss inverted microscope at 100X magnification. Panel A shows the wild type strain, Panel B the Δ*kdnase* knockout*,* and Panel C shows the *Δkdnase*^{R} strain that displays an intermediate phenotype. The deposits in the periphery of the plate in Panel A are dye precipitates.

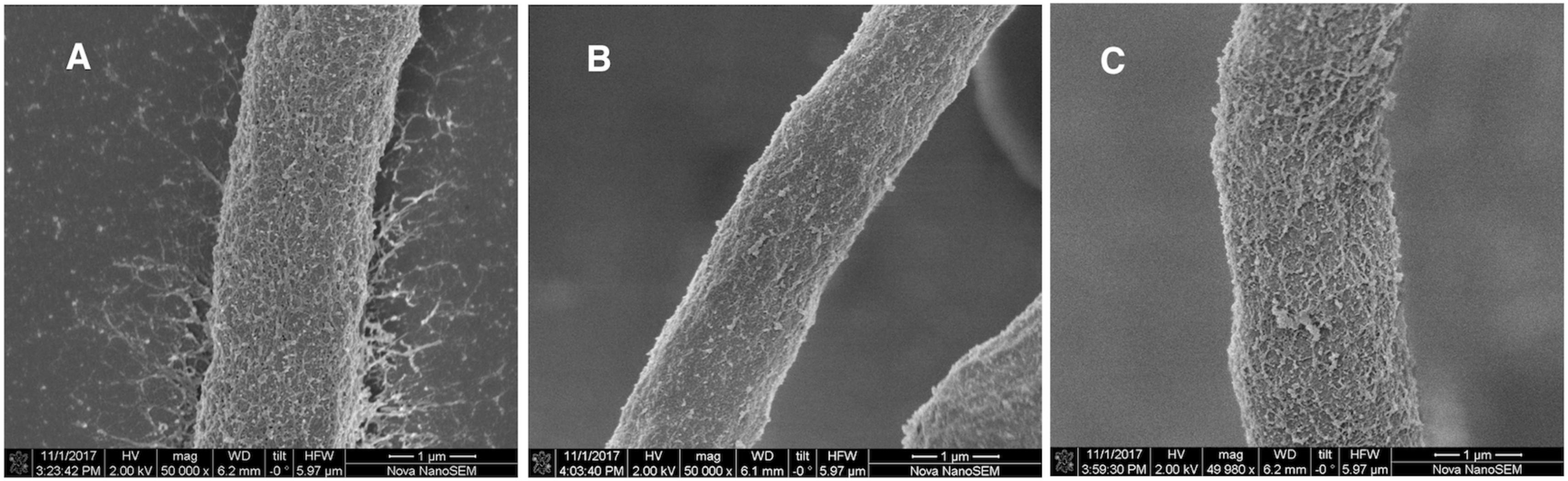

Figure S6. Scanning electron microscopy of *A. fumigatus* hyphae. Conidia were inoculated into buffered RPMI medium and grown on coverslips overnight for 24h at 37°C. Each image was taken at 50,000X magnification and a 1-µm scale bar is below each image. A. WT; B. *Δkdnase* and C. *Δkdnase*^{R}.

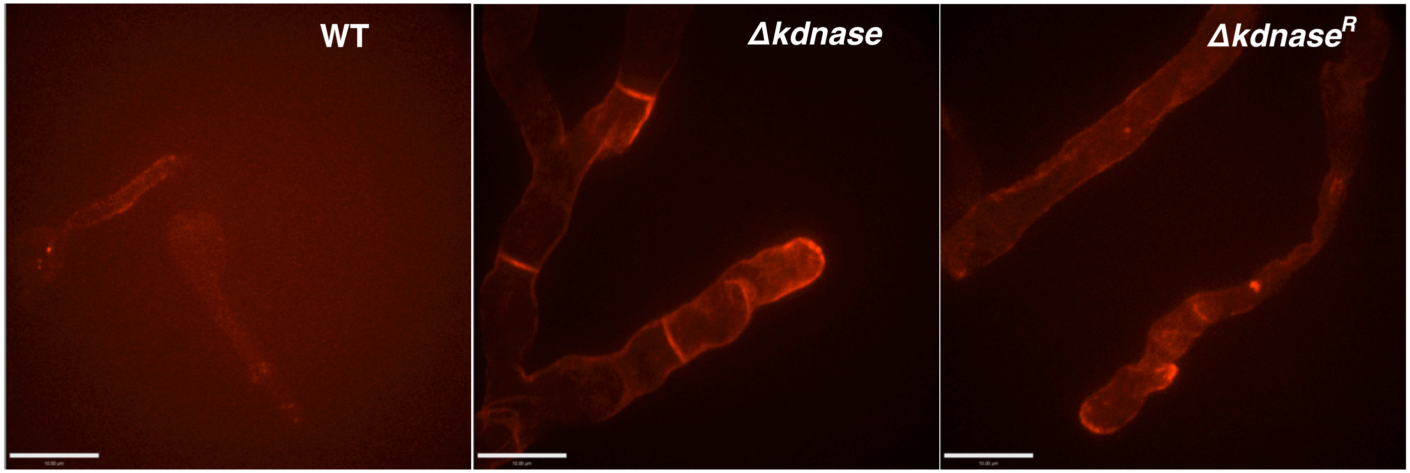

Figure S7. Representative images of a fluorescent chitin-binding probe in hyphae from the wild type, *Δkdnase* and *Δkdnase*^{R} strains. Cells were grown in YAG supplemented with 1M sorbitol. Chitin was detected using confocal microscopy using a fluorophore-conjugated chitin-binding probe (NEB). White bars represent 10 µm. Gain was increased for WT only to highlight hyphae for this image.

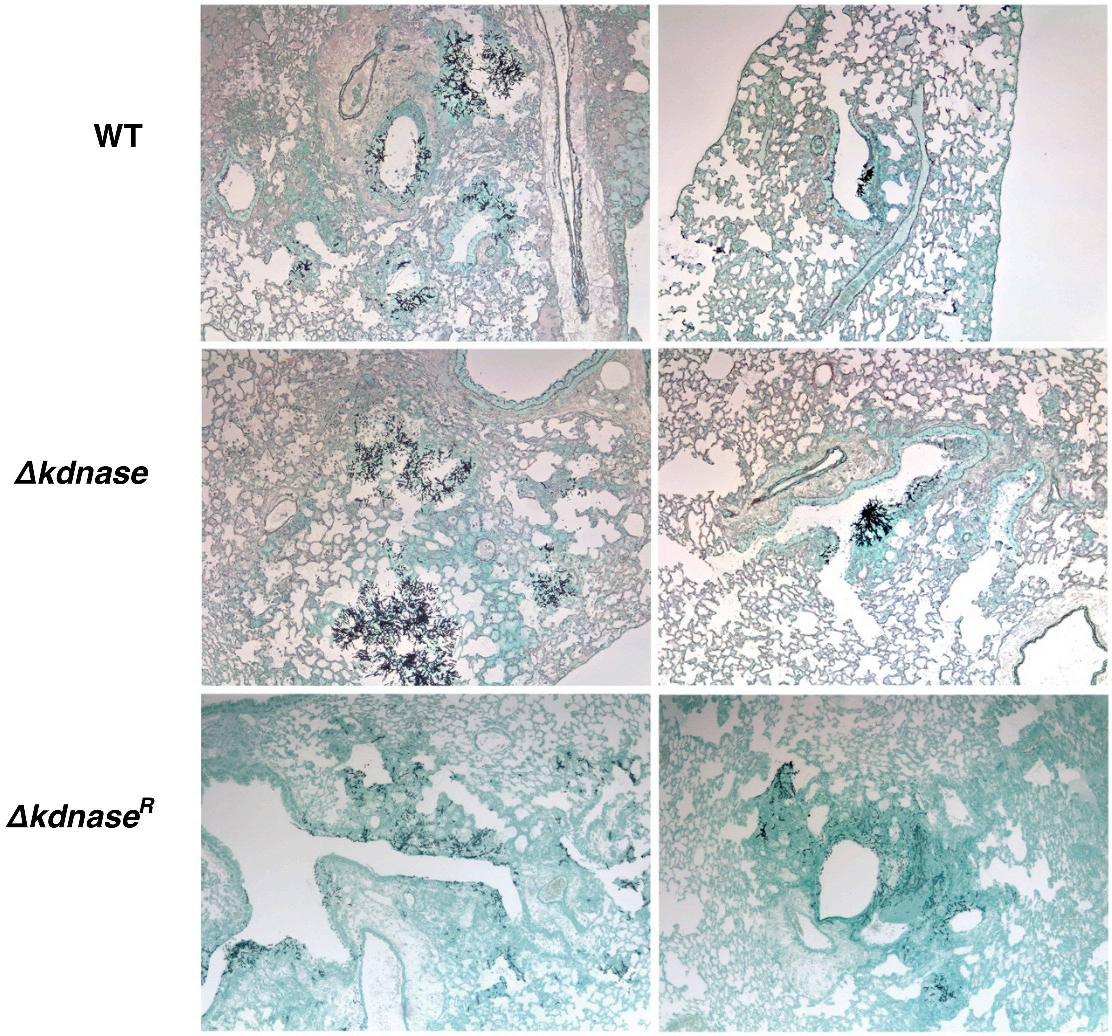

Figure S8. Representative images of GMS-stained lung sections from mice exposed to wild type (WT), *Δkdnase* (KO) and the *Δkdnase*^{R} (Rescued) strain with no AmBisome treatment. Fungal hyphae stain black. All mice died within 4 days of inoculation. Left and Right panels represent sections from different mice in each group; all images were captured at 100X magnification.

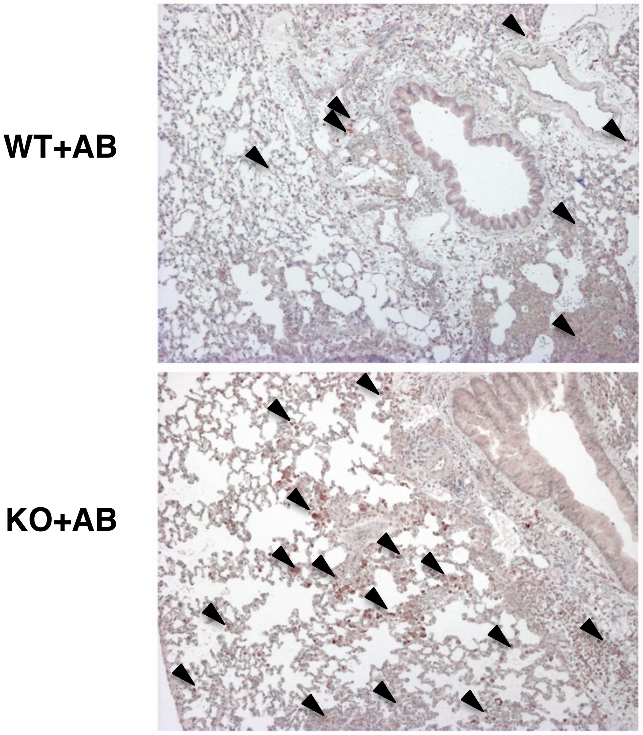

Figure S9. Representative Mac3+ stained images of sections from mice exposed to wild type (WT) or *Δkdnase* (KO) *A. fumigatus* conidia followed by AmBisome (AB) treatment. Images were captured at 100X magnification. Arrows point to a subset of the red cells that were considered Mac3+.
